# Nanoparticles (NPs)‐Meditated LncRNA AFAP1‐AS1 Silencing to Block Wnt/*β*‐Catenin Signaling Pathway for Synergistic Reversal of Radioresistance and Effective Cancer Radiotherapy

**DOI:** 10.1002/advs.202000915

**Published:** 2020-08-05

**Authors:** Zhuofei Bi, Qingjian Li, Xiaoxiao Dinglin, Ying Xu, Kaiyun You, Huangming Hong, Qian Hu, Wei Zhang, Chenchen Li, Yujie Tan, Ning Xie, Wei Ren, Chuping Li, Yimin Liu, Hai Hu, Xiaoding Xu, Herui Yao

**Affiliations:** ^1^ Guangdong Provincial Key Laboratory of Malignant Tumor Epigenetics and Gene Regulation Medical Research Center Sun Yat‐Sen Memorial Hospital Sun Yat‐Sen University Guangzhou 510120 P. R. China; ^2^ RNA Biomedical Institute Sun Yat‐Sen Memorial Hospital Sun Yat‐Sen University Guangzhou 510120 P. R. China; ^3^ Department of Oncology Sun Yat‐sen Memorial Hospital Sun Yat‐sen University Guangzhou 510120 P. R. China; ^4^ Breast Tumor Center Sun Yat‐sen Memorial Hospital Sun Yat‐sen University Guangzhou 510120 P. R. China

**Keywords:** cancer radiotherapy, lncRNAs, nanoparticles, radioresistance, Wnt/*β*‐catenin signaling pathway

## Abstract

Resistance to radiotherapy is frequently encountered in clinic, leading to poor prognosis of cancer patients. Long noncoding RNAs (lncRNAs) play important roles in the development of radioresistance due to their functions in regulating the expression of target genes at both transcriptional and posttranscriptional levels. Exploring key lncRNAs and elucidating the mechanisms contributing to radioresistance are crucial for the development of effective strategies to reverse radioresistance, which however remains challenging. Here, actin filament‐associated protein 1 antisense RNA1 (lncAFAP1‐AS1) is identified as a key factor in inducing radioresistance of triple‐negative breast cancer (TNBC) via activating the Wnt/*β*‐catenin signaling pathway. Considering the generation of a high concentration of reduction agent glutathione (GSH) under radiation, a reduction‐responsive nanoparticle (NP) platform is engineered for effective lncAFAP1‐AS1 siRNA (siAFAP1‐AS1) delivery. Systemic delivery of siAFAP1‐AS1 with the reduction‐responsive NPs can synergistically reverse radioresistance by silencing lncAFAP1‐AS1 expression and scavenging intracellular GSH, leading to a dramatically enhanced radiotherapy effect in both xenograft and metastatic TNBC tumor models. The findings indicate that lncAFAP1‐AS1 can be used to predict the outcome of TNBC radiotherapy and combination of systemic siAFAP1‐AS1 delivery with radiotherapy can be applied for the treatment of recurrent TNBC patients.

## Graphical abstract

Herein, lncAFAP1‐AS1 is demonstrated as a key factor in inducing radioresistance by activating the Wnt/*β*‐catenin signaling pathway. Considering the generation of a high concentration of glutathione (GSH) under radiation, a reduction‐responsive nanoplatform is engineered for effective siRNA delivery, which can synergistically reverse radioresistance by silencing lncAFAP1‐AS1 expression and scavenging intracellular GSH, leading to a dramatically enhanced cancer radiotherapy effect.

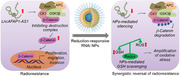

## 1 Introduction

Radioresistance is a frequently encountered issue in clinic, which commonly leads to enhanced local invasion, metastasis, and poor prognosis of cancer patients.^[^ ^1^, ^2^ ^]^ One important reason leading to radioresistance is self‐regulated intracellular redox homeostasis.^[^ ^3^, ^4^ ^]^ Ionizing radiation can induce the generation of high level of reactive oxygen species (ROS), such as hydroxyl radical (•OH) and superoxide radical (O_2_^−^) that can cause DNA double‐strand breaks or activate apoptotic signaling pathways to induce cell apoptosis.^[^ ^1^, ^3^, ^5^ ^]^ However, tumor cells usually adaptively generate a high concentration of antioxidants (e.g., superoxide dismutase and glutathione peroxidase) and reduction agents, such as glutathione (GSH), which can scavenge the elevated ROS and maintain the intracellular redox homeostasis for cell survival, ultimately leading to radioresistance.^[^ ^4^, ^6^ ^]^ Therefore, disrupting redox homeostasis via promoting ROS generation and/or reducing ROS scavenging has long been considered as an effective strategy to enhance radiosensitivity.^[^ ^5^, ^7^ ^]^ However, radioresistance is a complex process associated with multiple biological changes (e.g., evasion of apoptosis, altered DNA damage response, enhanced DNA repair, etc.) that are regulated by intrinsic cell signaling networks.^[^ ^8^ ^]^ Therefore, beneficial treatment for radioresistant cancer patients needs to include comprehensive consideration of the multiple biological changes contributing to radioresistance.

Long noncoding RNAs (lncRNAs) are a class of noncoding transcripts that are widely involved in tumorigenesis and progression, and regulation of their expression usually induces multiple biological changes.^[^ ^9^, ^10^ ^]^ Due to their important role in the intrinsic cell signaling networks, lncRNAs have been recently identified as attractive therapeutic targets for effective cancer treatment. Currently, series of functional lncRNAs have been identified as key factors to induce radioresistance by regulating the expression of target genes at transcriptional or post‐transcriptional levels.^[^ ^11^, ^12^ ^]^ Although the regulatory mechanisms of these lncRNAs have been extensively elucidated, their clinical translation is significantly hindered because few efforts have been focused on developing effective in vivo delivery strategies to regulate the biological functions of lncRNAs. In addition, compared to the billions of intracellular lncRNAs that may exert their biological functions,^[^ ^13^ ^]^ the proportion of currently discovered radioresistance‐associated lncRNAs is still very low and thus more functional lncRNAs and their regulatory mechanisms need to be explored.

Here, we utilized locally recurrent tumors of triple‐negative breast cancer (TNBC) patients and identified a key lncRNA (actin filament‐associated protein 1 antisense RNA1, AFAP1‐AS1), which plays an important role in regulating radiosensitivity of TNBC via activating the canonical Wnt/*β*‐catenin signaling pathway. Based on this regulatory mechanism, a nanoparticle (NP)‐based small interference RNA (siRNA) delivery platform was used to transport lncAFAP1‐AS1 siRNA (siAFAP1‐AS1) for the reversal of radioresistance. Moreover, considering the generation of a high concentration of GSH under radiation,^[^ ^6^ ^]^ a reduction‐responsive poly(disulfide amide) (PDSA) polymer was encapsulated into the NP platform,^[^ ^14^, ^15^ ^]^ which is expected to enhance the reversal of radioresistance via concurrently silencing lncAFAP1‐AS1 expression and scavenging intracellular GSH. After systemic administration, the siRNA loaded NPs could accumulate in the tumor sites via the enhanced permeability and retention effect.^[^ ^16^ ^]^ Subsequently, the high concentration of intracellular GSH could destroy the NP structure,^[^ ^17^, ^18^ ^]^ thus leading to concurrent scavenging of intracellular GSH and fast release of siAFAP1‐AS1 for efficient gene silencing. With this synergistic reversal of radioresistance, systemic delivery of siAFAP1‐AS1 with the reduction‐responsive NP platform could dramatically improve the radiotherapy effect in both xenograft and metastatic tumor models.

## 2 Results

### 2.1 LncAFAP1‐AS1 Expression is Upregulated in Radioresistant TNBC Patients and Indicates Poor Outcome and Resistance to Radiotherapy

**Figure 1:**
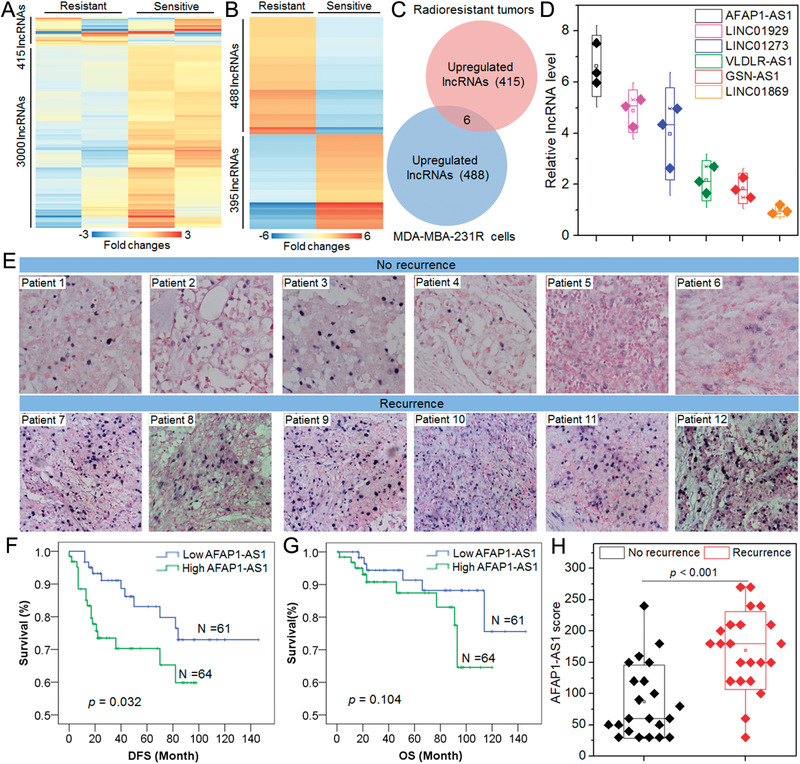
LncAFAP1‐AS1 expression is upregulated in radioresistant TNBC patients and indicates poor outcome and resistance to radiotherapy. A) The heatmap of lncRNAs that are upregulated or downregulated over threefolds in the matched tumor tissues of two TNBC patients before receiving postoperative radiotherapy (radiosensitive) and their locally recurrent tumor tissues after receiving postoperative radiotherapy (radioresistant). B) The heatmap of lncRNAs that are upregulated or downregulated at least sixfold in MDA‐MB‐231R cells comparing to that in the parental MDA‐MB‐231 cells. C) The number of overlapped lncRNAs that are upregulated in locally recurrent tumor tissues of radioresistant patients and MDA‐MB‐231R cells. The threshold is threefold differentially expressed in recurrent tumor tissues and sixfold differentially expressed in MDA‐MB‐231R cells. D) qRT‐PCR analysis of the expression level of the overlapped lncRNAs shown in (C) in MDA‐MB‐231R cells. E) ISH images of the lncAFAP1‐AS1 expression in tumor tissues of TNBC patients without (*n* = 6) or with recurrence (*n* = 6) after receiving postoperative radiotherapy. F) DFS and G) OS of TNBC patients (*n* = 125) with different lncAFAP1‐AS1 expression levels in their tumors. H) LncAFAP1‐AS1 score determined by ISH assay in the tumor tissues of TNBC patients without (*n* = 22) or with recurrence (*n* = 22) after receiving postoperative radiotherapy.

To explore the key lncRNA that regulates the radiosensitivity of TNBC, we first collected the matched tumor tissues of two TNBC patients (Table S1, Supporting Information) before postoperative radiotherapy (surgically resected tumors, denoted radiosensitive) and after postoperative radiotherapy with local recurrence (core needle biopsies of recurrent tumors, denoted radioresistant). Then, we employed lncRNA microarray to analyze and compare their lncRNA expression profiles (**Figure** **1**A). Moreover, in order to explore the molecular mechanism that induces the radioresistance of TNBC, we further established a radioresistant TNBC cell line (MDA‐MB‐231R, Figure S1, Supporting Information) and also compared its lncRNA expression profile with the parental MDA‐MB‐231 cell line (Figure 1B). As shown in Figure 1C, six lncRNAs are upregulated in both radioresistant tumor samples and MDA‐MB‐231R cells. We further validated their high expression in MDA‐MB‐231R cells (Figure 1D) using reverse transcription quantitative polymerase chain reaction (qRT‐PCR). Among these upregulated lncRNAs, lncAFAP1‐AS1 is particularly noted, because this lncRNA is not only upregulated in the radioresistant cells and tumor samples, but also its high expression is correlated with low survival rate of TNBC patients in the Cancer Genome Atlas (TCGA) database (Figure S2, Supporting Information).

To further validate the high expression of lncAFAP1‐AS1 in radioresistant TNBC, in situ hybridization (ISH) was performed using the surgically resected tumor tissues of TNBC patients (*n* = 12) receiving postoperative radiotherapy (Figure 1E). Compared to the patients (*n* = 6) without recurrence, higher lncAFAP1‐AS1 expression can be found in the tumor tissues of the patients (*n* = 6) with local recurrence. More importantly, through examining lncAFAP1‐AS1 expression in the tumor tissues of 125 TNBC patients (Table S2, Supporting Information), its high expression is correlated with poor disease‐free survival (DFS) (Figure 1F) and overall survival (OS) (Figure 1G). In addition, within these 125 TNBC patients, lncAFAP1‐AS1 expression is obviously higher in the tumor tissues of patients with local recurrence compared to the patients without recurrence (Figure 1H). All these results strongly demonstrate that high lncAFAP1‐AS1 expression is associated with radioresistance of TNBC patients, and the expression level of this lncRNA in tumor tissues could be used to predict the outcome of TNBC radiotherapy.

### 2.2 LncAFAP1‐AS1 Induces Radioresistance of TNBC via Activating the Wnt/*β*‐catenin Signaling Pathway

**Figure 2:**
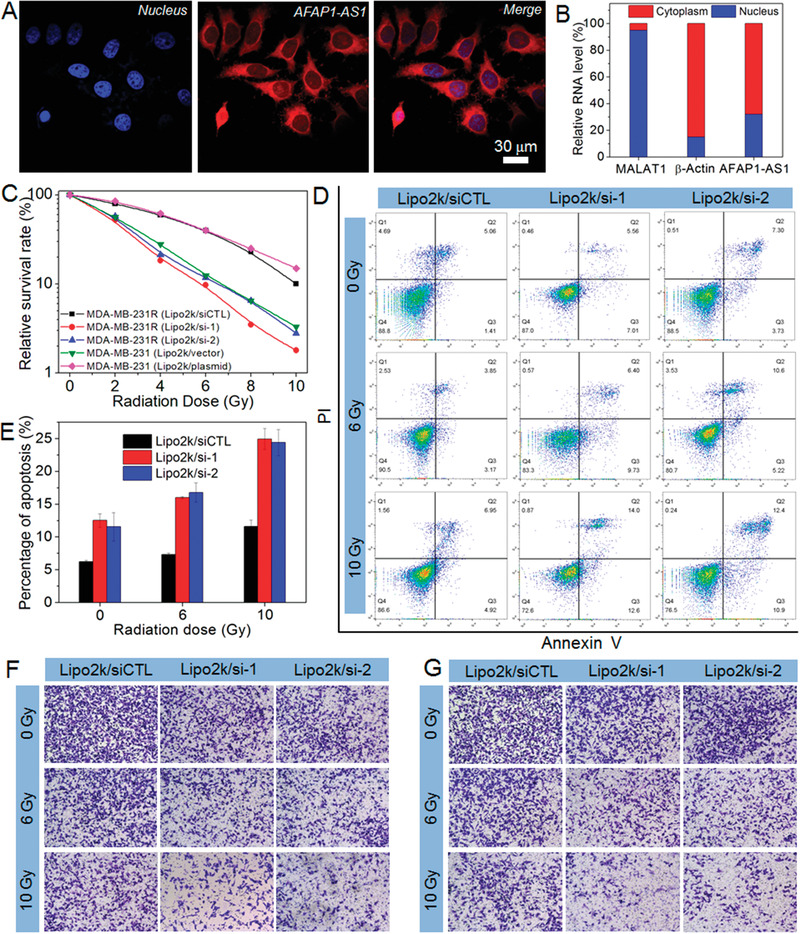
LncAFAP1‐AS1 is predominately located in the cytoplasm and its high expression promotes TNBC cell proliferation, migration, and invasion. A) FISH images of MDA‐MB‐231R cells observed under CLSM. B) LncAFAP1‐AS1 expression in the cytoplasm and nuclei of MDA‐MB‐231R cells determined by nuclear/cytoplasm fractionation. C) Relative survival rate of MDA‐MB‐231 cells treated with the Lipo2k/plasmid complexes at a plasmid concentration of 1.5 µg mL^−1^ followed by different doses of X‐ray radiation or MDA‐MB‐231R cells treated with the Lipo2k/si‐1, Lipo2k/si‐2, or Lipo2k/siCTL complexes at a siRNA concentration of 30 × 10^−9^ m followed by different doses of X‐ray radiation. D) Flow cytometry profile and E) percentage of apoptosis of MDA‐MB‐231R cells treated with the formulas in (C) followed by different doses of X‐ray radiation. F) Migration and G) invasion of MDA‐MB‐231R cells treated with the Lipo2k/si‐1 or Lipo2k/si‐2 complexes at a siRNA dose of 15 × 10^−9^ m followed by different doses of X‐ray radiation.

Having validated the high lncAFAP1‐AS1 expression in radioresistant TNBC, we next explored how this lncRNA induces radioresistance. Fluorescence in situ hybridization (FISH) was used to examine the location of lncAFAP1‐AS1 and the result shows that this lncRNA is predominately located in the cytoplasm (**Figure** **2**A). This result is further confirmed by nuclear/cytoplasm fractionation (Figure 2B). Using 5′ and 3′‐rapid amplification of cDNA ends, lncAFAP1‐AS1 is found to be a 6795 nucleotide (nt) transcript that is identical to NONHSAG037420 in GeneCards or ENST00000608442.1 in the UCSC database. With this information, we next evaluated the biological function of lncAFAP1‐AS1 via silencing its expression in MDA‐MB‐231R cells or upregulating its expression in the parental MDA‐MB‐231 cells (Figure S3, Supporting Information). Silencing lncAFAP1‐AS1 expression using the complexes of lipofectamine 2000 and siAFAP1‐AS1 sequence 1 or 2 (Lipo2k/si‐1 or Lipo2k/si‐2) at a siRNA dose of 30 × 10^−9^ m apparently enhances the radiosensitivity of MDA‐MBA‐231R cells, including low cell viability (Figure 2C) and more apoptosis (Figure 2D,E). In addition, with the lncRNA silencing at a siRNA dose of 15 × 10^−9^ m that shows efficient gene silencing but does not induce apparent apoptosis (Figure S3, Supporting Information), MDA‐MBA‐231R cells show weakened migration (Figure 2F) and invasion (Figure 2G) abilities under radiation. In contrast, upregulating lncAFAP1‐AS1 expression using the complexes of Lipo2k and the plasmid of lncAFAP1‐AS1 (Lipo2k/plasmid) could induce the radioresistance of parental MDA‐MBA‐231 cells and thus the resulting cells maintain relatively high viability under radiation (Figure 2C).

**Figure 3:**
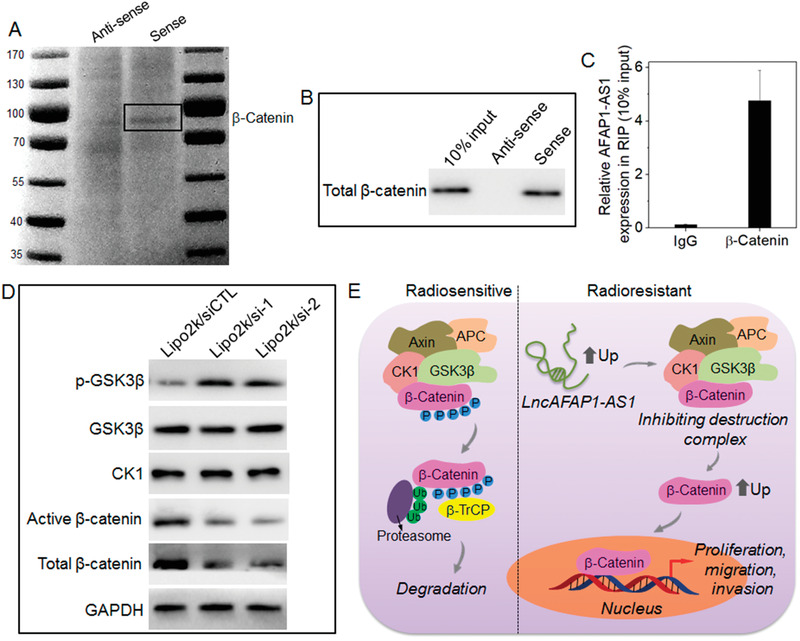
LncAFAP1‐AS1 induces radioresistance of TNBC via activating the Wnt/*β*‐catenin signaling pathway. A) The silver staining and B) Western blot analysis of the RNA‐protein binding complexes after RNA pulldown of lncAFAP1‐AS1 in MDA‐MB‐231R cells. C) RIP of *β*‐catenin in MDA‐MB‐231R cells. LncAFAP1‐AS1 was retrieved by *β*‐catenin or IgG and then detected by qRT‐PCR. D) Western blot analysis of the expression of CK1, GSK3*β*, p‐GSK3*β*, and active and total *β*‐catenin in MDA‐MBA‐231R cells treated with the Lipo2k/si‐1, Lipo2k/si‐2, or Lipo2k/siCTL complexes at a siRNA concentration of 30 × 10^−9^ m. E) Schematic illustration of the molecular mechanism of lncAFAP1‐AS1 to induce TNBC radioresistance via suppressing the function of *β*‐catenin destruction complex and subsequently activating the Wnt/*β*‐catenin signaling pathway to promote TNBC cell proliferation, migration, and invasion.

After evaluating the biological function of lncAFAP1‐AS1, we next explored its molecular mechanism in regulating TNBC radiosensitivity. To this end, RNA pulldown followed by mass spectrometry (MS) was used to identify the proteins interacting with lncAFAP1‐AS1 since the majority of lncRNAs exert their biological functions via interacting with their counterpart proteins.^[^ ^9^ ^]^ As shown in **Figure** **3**A, one protein band (85–95 kDa) can be specifically enriched in the lncAFAP1‐AS1 pulldown complexes. Further MS analysis shows that *β*‐catenin is the specific lncAFAP1‐AS1 binding protein (Figure S4, Supporting Information). The specific interaction between lncAFAP1‐AS1 and *β*‐catenin is further validated by RNA pulldown assay followed by Western blot analysis (Figure 3B), RNA immunoprecipitation (RIP) using antibody against *β*‐catenin (Figure 3C), and colocalization between lncAFAP1‐AS1 and *β*‐catenin after staining intracellular lncAFAP1‐AS1 and *β*‐catenin with different fluorescent probes (Figure S5, Supporting Information). Moreover, by using truncated lncAFAP1‐AS1 containing 1–60 nt with the possible ability to bind *β*‐catenin, the sequence nt 3561–3620 of lncAFAP1‐AS1 is identified as the binding site of *β*‐catenin (Figure S6, Supporting Information). The dissociation constant (*K*_d_) between lncAFAP1‐AS1 and *β*‐catenin could be calculated as ≈3.59 × 10^−9^ m using RNA pulldown assay followed by Western blot analysis (Figure S7, Supporting Information). *β*‐Catenin is a classical transcription factor and key effector of the oncogenic Wnt signaling pathway.^[^ ^19^ ^]^ Wnt activation can promote the entrance and accumulation of *β*‐catenin in the nucleus to form a transcription complex with T cell‐specific transcription factor/LEF‐1, subsequently activating the transcription of downstream target genes to promote cell proliferation, metastasis, and invasion.^[^ ^20^, ^21^, ^22^ ^]^ In mammalian cells, *β*‐catenin expression is tightly controlled by the destruction complex, in which the tumor suppressor protein Axin acts as a scaffold to interact with *β*‐catenin, adenomatous polyposis coli (APC), casein kinase 1 (CK1), and glycogen synthase kinase 3*β* (GSK3*β*).^[^ ^23^, ^24^ ^]^ In this destruction complex, CK1 and phosphorylated GSK3*β* (p‐GSK3*β*) can induce the phosphorylation of Axin‐bound *β*‐catenin, leading to *β*‐catenin ubiquitination by *β*‐transducin repeat‐containing protein (*β*‐TrCP) and rapid degradation by proteasome.^[^ ^25^ ^]^ Therefore, we predict that lncAFAP1‐AS1 might suppress the function of the *β*‐catenin destruction complex and thus induce the release of *β*‐catenin to exert its biological function. To verify our prediction, we silenced lncAFAP1‐AS1 expression and then examined the expression of CK1, GSK3*β*, p‐GSK3*β*, and *β*‐catenin. As shown in Figure 3D, MDA‐MBA‐231R cells with high lncAFAP1‐AS1 expression show low level of GSK3*β* phosphorylation and high expression of total and active *β*‐catenin. After silencing lncAFAP1‐AS1 expression, there is no obvious change in the expression of CK1 and GSK3*β*, but the p‐GSK3*β* expression is significantly improved. With this upregulated p‐GSK3*β* expression to induce *β*‐catenin phosphorylation and subsequent ubiquitination (Figure S8, Supporting Information), there is a dramatic decrease in the expression of total and active *β*‐catenin. This result indicates that lncAFAP1‐AS1 can block the function of *β*‐catenin destruction complex by suppressing p‐GSK3*β* expression, thereby leading to the release of *β*‐catenin and activating the Wnt/*β*‐catenin signaling pathway to promote TNBC cell proliferation, migration, and invasion (Figure 3E).

### 2.3 Reduction‐Responsive NPs‐Mediated lncAFAP1‐AS1 Silencing Synergistically Reverses Radioresistance

**Figure 4:**
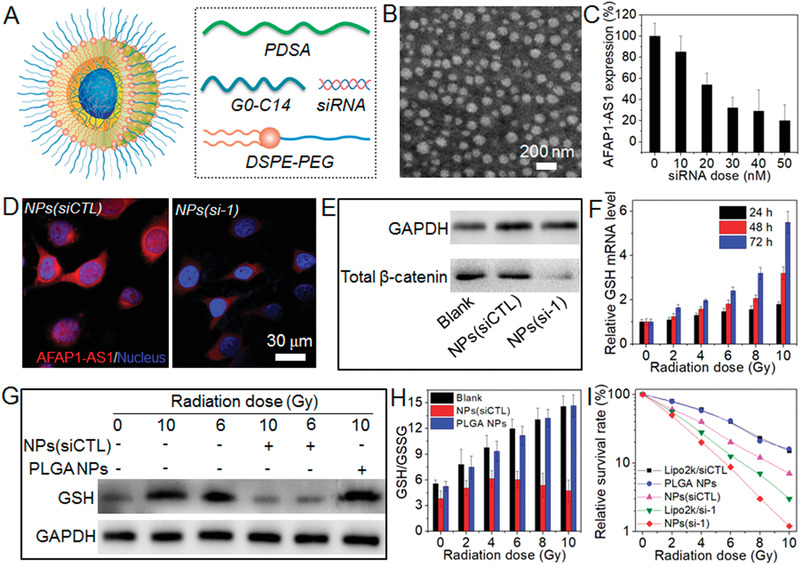
Reduction‐responsive NPs‐mediated lncAFAP1‐AS1 silencing synergistically reverses TNBC radioresistance. A) Schematic illustration of the NPs(si‐1) made with the PDSA polymer, lipid‐PEG (1,2‐distearoyl‐*sn*‐glycero‐3‐ phosphoethanolamine‐*N*‐[methoxy (poly ethylene glycol)‐3000], DSPE‐PEG_3k_), and cationic lipid G0‐C14. B) Morphology of the NPs(si‐1) dispersed in aqueous solution. C) LncAFAP1‐AS1 expression level determined by qRT‐PCR in MDA‐MBA‐231R cells treated with the NPs(si‐1) at different siRNA doses. D) FISH images of MDA‐MB‐231R cells treated with the NPs(siCTL) or NPs(si‐1) at a siRNA dose of 30 × 10^−9^ m. E) Western blot analysis of *β*‐catenin expression in MDA‐MB‐231R cells treated with the NPs(siCTL) or NPs(si‐1) at a siRNA dose of 30 × 10^−9^ m. F) qRT‐PCR analysis of GSH mRNA level in MDA‐MB‐231 cells at 24, 48, and 72 h post different doses of X‐ray radiation. G) Western blot analysis of GSH expression and H) intracellular GSH/GSSG ratio in MDA‐MB‐231 cells treated with the NPs(siCTL) or PLGA NPs for 24 h at a siRNA concentration of 30 × 10^−9^ m followed by different does of X‐ray radiation. I) Relative survival rate of MDA‐MB‐231R cells treated with the Lipo2k/si‐1 complexes, Lipo2/siCTL complexes, NPs(siCTL), NPs(si‐1), or PLGA NPs at a siRNA concentration of 30 × 10^−9^ m followed by different doses of X‐ray radiation.

Since lncAFAP1‐AS1 can activate the Wnt/*β*‐catenin signaling pathway to induce the radioresistance of TNBC, systemic delivery of siRNA to silence lncAFAP1‐AS1 expression could be a promising strategy to reverse radioresistance. In the past decade, NPs have been demonstrated as powerful tools for systemic siRNA delivery and disease treatment.^[^ ^26^, ^27^, ^28^ ^]^ In particular, because tumor microenvironment or tumor cells show several distinguishing biological/endogenous factors (e.g., pH, enzymes, redox, and hypoxia) compared to normal tissues or cells,^[^ ^29^ ^]^ bioresponsive NPs have been recently developed as effective siRNA delivery carriers, which can respond to biological stimuli to achieve efficient intracellular siRNA delivery and better anticancer effect.^[^ ^30^ ^]^ Here, because tumor cells usually adaptively generate a high concentration of GSH that can scavenge the elevated ROS under radiation and maintain intracellular redox homeostasis,^[^ ^6^ ^]^ we employed the reduction‐responsive NPs we previously developed for systemic siAFAP1‐AS1 delivery (Figure S9, Supporting Information),^[^ ^15^ ^]^ which is expected to synergistically reverse radioresistance via concurrently silencing lncAFAP1‐AS1 expression and disrupting intracellular redox homeostasis. This NP platform is composed of a solid poly(disulfide amide) (PDSA)/cationic lipid core and a lipid‐poly(ethylene glycol) (lipid‐PEG) shell (**Figure** **4**A). In addition, because both siAFAP1‐AS1 sequence 1 and 2 show the similar lncAFAP1‐AS1 silencing efficacy (Figure S3, Supporting Information), we encapsulated sequence 1 into the reduction‐responsive NPs with the optimal gene silencing (denoted NPs(si‐1), Figure S9, Supporting Information). As shown in Figure 4B, the siRNA loaded NPs show a uniform spherical morphology  with an average size of ≈110 nm (Figure S10, Supporting Information) and siRNA encapsulation efficiency of ≈60%. For this NP platform, due to the presence of multiple disulfide bonds in the structure of the PDSA polymer (Figure S11, Supporting Information), it shows a favorable reduction response, as demonstrated by the increased particle size, destroyed nanostructure, and fast siRNA release upon the addition of GSH (Figure S12, Supporting Information). With this favorable reduction response, the NPs(si‐1) could efficiently silence lncAFAP1‐AS1 expression in MDA‐MBA‐231R cells and more than 70% of lncAFAP1‐AS1 expression could be suppressed at a siRNA dose of 30 × 10^−9^ m (Figure 4C). The weak red fluorescence in FISH assay (Figure 4D) also demonstrates the efficient gene silencing of the NPs(si‐1). With this efficient lncAFAP1‐AS1 silencing, *β*‐catenin expression is significantly blocked (Figure 4E), proving that lncAFAP1‐AS1 exerts its biological function via activating the Wnt/*β*‐catenin signaling pathway.

Having confirmed the efficient gene silencing, we next examined whether the NPs(si‐1) can use their redox response to scavenge intracellular GSH and disrupt redox homeostasis. In order to avoid the potential influence of lncAFAP1‐AS1 silencing on GSH scavenging, reduction‐responsive NPs loaded with control siRNA (denoted NPs(siCTL)) were used to treat the TNBC cells. As shown in Figure 4F,G, MDA‐MBA‐231 cells indeed generate high level of GSH under radiation, similar to the high GSH expression in MDA‐MBA‐231R cells (Figure S13, Supporting Information). Moreover, this elevated GSH expression leads to the increased ratio between reduced glutathione and oxidized glutathione (GSH/GSSG) (Figure 4H). However, when using the NPs(siCTL) to treat MDA‐MBA‐231 cells, the GSH expression always maintains low level even under radiation (Figure 4G) and there is nearly no change in the intracellular GSH/GSSG ratio compared to the cells without radiation treatment (Figure 4H). This result clearly indicates that the NPs(siCTL) can scavenge GSH and disrupt intracellular redox homeostasis. To verify the contribution of the reduction response to GSH scavenging, commercially available poly(lactic‐*co*‐glycolic acid) (PLGA) with a similar molecular weight (viscosity, 0.1–0.25 dL g^−1^) as that of the PDSA polymer was used to prepare nonresponsive PLGA NPs and then incubated with MDA‐MBA‐231 cells. Due to the absence of reduction response, high level of GSH expression can be found in the cells (Figure 4G) and the intracellular GSH/GSSG ratio is much higher compared to the cells without radiation treatment (Figure 4H).

**Figure 5:**
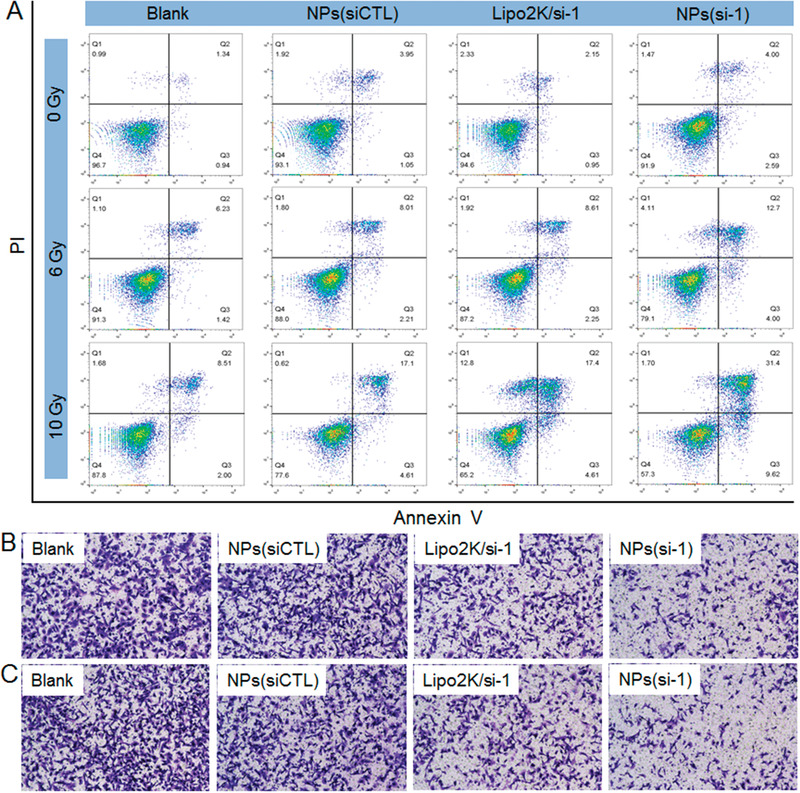
Reduction‐responsive NPs‐mediated lncAFAP1‐AS1 silencing enhances TNBC radiosensitivity and inhibits TNBC cell proliferation, migration, and invasion. A) Flow cytometry profiles of MDA‐MB‐231R cells treated with the NPs(siCTL), Lipo2k/si‐1 complexes, or NPs(si‐1) at a siRNA concentration of 30 × 10^−9^ m followed by different doses of X‐ray radiation. B) Migration and C) invasion of MDA‐MB‐231R cells treated with the formulas in (A) at a siRNA concentration of 30 × 10^−9^ m followed by 10 Gy of X‐ray radiation.

With the above demonstrated ability to silence lncAFAP1‐AS1 expression and scavenge GSH, the NPs(si‐1) could synergistically reverse the radioresistance of MDA‐MBA‐231R cells. As shown in Figure 4I, the NPs(si‐1) is the most effective in inhibiting the survival of MDA‐MBA‐231R cells under radiation compared to the single GSH scavenging by NPs(siCTL) or single lncAFAP1‐AS1 silencing by Lipo2k/si‐1 complex. Flow cytometry analysis also shows the similar tendency (**Figure** **5**A). MDA‐MBA‐231R cells treated with the NPs(si‐1) show more apoptosis under radiation compared to the cells treated with the NPs(siCTL) or Lipo2k/si‐1 complex (Figure S14, Supporting Information). In addition, with the concurrent lncAFAP1‐AS1 silencing and GSH scavenging, MDA‐MBA‐231R cells also show much less migration (Figure 5B) and invasion (Figure 5C) under radiation.

### 2.4 Reduction‐Responsive NPs‐Mediated lncAFAP1‐AS1 Silencing Enhances Radiotherapy Effect in Xenograft and Metastatic Tumor Models

**Figure 6:**
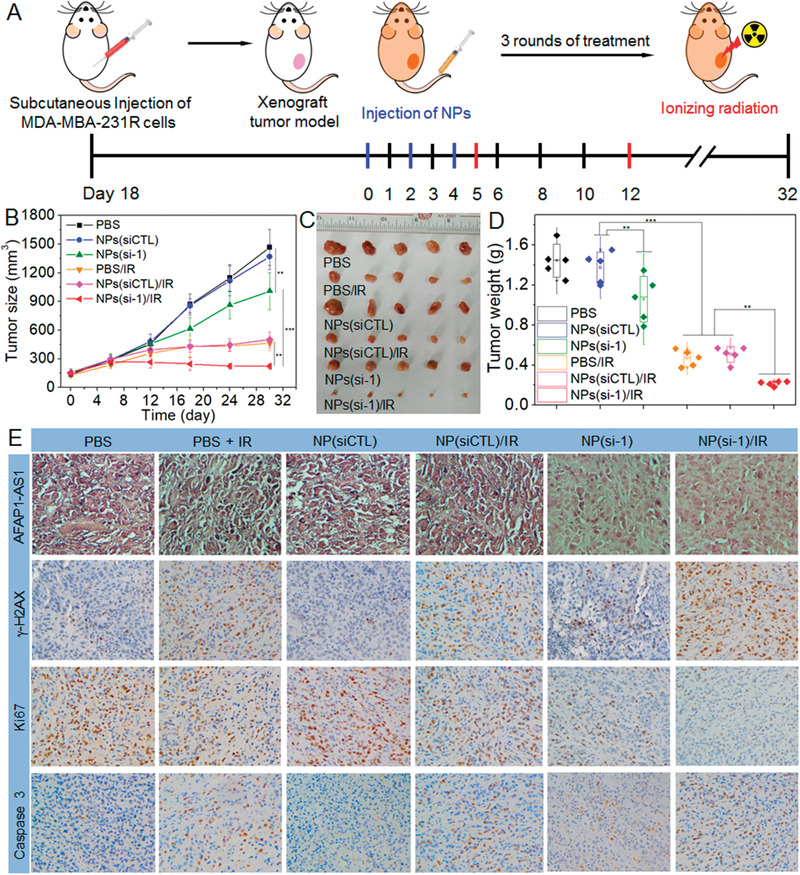
Reduction‐responsive NPs‐mediated lncAFAP1‐AS1 silencing enhances the radiotherapy effect in xenograft tumor model. A) Schematic illustration of tumor inoculation and different treatments in MDA‐MB‐231R tumor‐bearing nude mice. 18 days after tumor inoculation, mice were treated with PBS, PBS followed by ionizing radiation (PBS/IR), NPs(siCTL), NPs(siCTL) followed by ionizing radiation (NPs(siCTL)/IR), NPs(si‐1), or NPs(si‐1) followed by ionizing radiation (NPs(si‐1)/IR) at a 1 nmol siRNA dose per mouse and 10 Gy radiation dose per mouse. B) Tumor growth, C) image of collected tumors, and D) tumor weight of the MDA‐MB‐231R tumor‐bearing mice treated with the formulas in (A). **p < 0.01, ***p < 0.001. E) LncAFAP1‐AS1 expression determined by ISH and the expression of *γ*H2AX, Ki67, and caspase 3 determined by IHC in the MDA‐MB‐231R tumors collected at the end point (Day 30).

With the promising in vitro results described above, we finally evaluated whether the NPs(si‐1) could enhance the therapeutic outcome of radiotherapy since they show prolonged blood circulation and high tumor accumulation (Figure S15, Supporting Information). To this end, the NPs(si‐1) were intravenously injected into the MDA‐MBA‐231R xenograft tumor‐bearing mice once every 2 days at a 1 nmol siRNA dose per mouse (*n* = 5) (**Figure** **6**A). After three consecutive injections, the tumors show favorable sensitivity to radiation and tumor growth is significantly inhibited compared to the mice treated with phosphate buffered saline (PBS), or NPs(siCTL) (Figure 6B–D; and Figure S16, Supporting Information). Within a long evaluation period of 30 days, there is less than 1.5‐fold increase (from ≈150 to ≈220 mm^3^) in the tumor size of the mice treated with the NPs(si‐1) followed by radiation (Figure 6B). In contrast, for the mice treated with PBS or NPs(siCTL) followed by radiation, they show more than 3.5‐fold increase in their tumor size and tumor weight. The immunohistochemistry (IHC) staining of tumor slides also indicates that the NPs(si‐1) treatment followed by radiation is the most effective in inhibiting tumor growth, as demonstrated by low lncAFAP1‐AS1 expression, more DNA damage (*γ*H2AX) and apoptosis (caspase 3), and less proliferation (Ki67) (Figure 6E; and Figure S17, Supporting Information).

**Figure 7:**
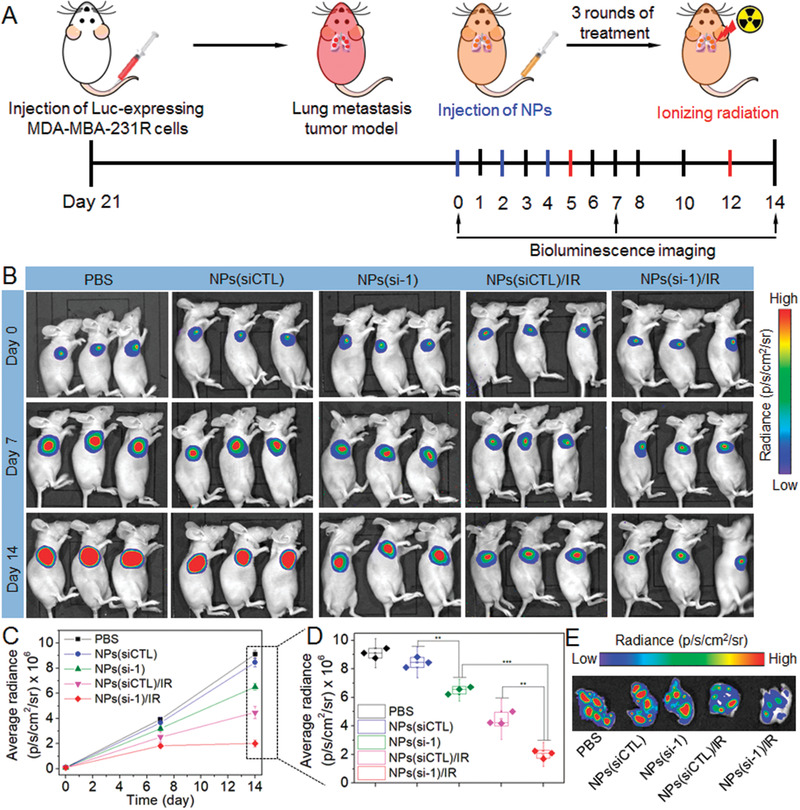
Reduction‐responsive NPs‐mediated lncAFAP1‐AS1 silencing enhances radiotherapy effect in metastatic tumor model. A) Schematic illustration of tumor inoculation and different treatments in the Luc‐MDA‐MB‐231R metastatic tumor‐bearing nude mice. 21 days after tumor inoculation, mice were treated with PBS, NPs(siCTL), NPs(siCTL) followed by ionizing radiation (NPs(siCTL)/IR), NPs(si‐1),or NPs(si‐1) followed by ionizing radiation (NPs(si‐1)/IR) at a 1 nmol siRNA dose per mouse and10 Gy radiation dose per mouse. B) Bioluminescence images of the Luc‐MDA‐MB‐231R metastatic tumor‐bearing nude mice at day 0, 7, and 14. C) Average radiance of tumor burden determined by bioluminescence imaging at day 0, 7, and 14. D) Average radiance of tumor burden at the endpoint (Day 14). **p < 0.01, ***p < 0.001. E) Bioluminescence images of the metastatic tumor tissues in the lung collected from the Luc‐MDA‐MB‐231R metastatic tumor‐bearing nude mice at the endpoint (Day 14).

Radioresistance usually leads to enhanced local invasion and metastasis of cancer patients. Therefore, we established a metastatic model using luciferase (Luc)‐expressing MDA‐MBA‐231R cells to further validate the ability of NPs(si‐1) to enhance the radiotherapy effect. Tumor growth was monitored by examining the average radiance of the tumor sites using bioluminescence imaging (**Figure** **7**A). As shown in Figure 7B–D, with the concurrent lncAFAP1‐AS1 silencing and GSH scavenging, the NPs(si‐1) treatment followed by radiation shows the strongest ability to reduce the metastatic tumor burden. After three rounds of treatments with the NPs and two rounds of radiation, all the mice were euthanized and their lungs were collected for metastasis analysis using bioluminescence imaging (Figure 7E). The result also indicates that the combination of NPs(si‐1) and radiation is the most effective in inhibiting the progression of the metastatic tumors.

It is noted that the administration of NPs(si‐1) shows neglectable in vivo side effects and no obvious influence on mouse body weight in both xenograft and metastatic models (Figure S18, Supporting Information). To further evaluate the potential in vivo side effects, the NPs were intravenously injected into normal adult mice (1 nmol siRNA dose per mouse, *n* = 3). Blood serum analysis shows that TNF‐*α*, IFN‐*γ*, IL‐6, and IL‐12 levels are in the normal range (Figure S19, Supporting Information). After three daily injections, multiple hematological parameters including aspartate aminotransferase, alanine aminotransferase, albumin, alkaline phosphatase, creatinine, globulin, total protein, and urea are in the normal range (Figure S20, Supporting Information). Histological analysis shows that there are no noticeable histological changes in the tissues from heart, liver, spleen, lung, or kidney (Figure S21, Supporting Information). All these results indicate the good biocompatibility of the NPs(si‐1) used in this work.

## 3 Discussion

Radiotherapy is an effective treatment for many cancers but radioresistance is frequently encountered. A recent clinical study shows that ≈18% of early stage breast cancer patients who received postoperative radiotherapy develop recurrence within 15 years.^[^ ^31^ ^]^ Radioresistance is a complex process associated with multiple signaling pathways. Among them, the Wnt/*β*‐catenin signaling pathway plays an extremely important role because it is a prosurvival pathway that can facilitate extensive crosstalk with other signaling pathways (e.g., phosphatidylinositol 3‐kinase/protein kinase B, PI3K/Akt).^[^ ^19^, ^21^, ^24^ ^]^ In fact, besides regulating radiosensitivity, this signaling pathway is widely involved in tumorigenesis and progression and its activation usually induces recurrence and metastasis of many types of cancers (e.g., colorectal, endometrial, gastric, lung, prostate, and breast cancer).^[^ ^20^, ^21^ ^]^ Therefore, the Wnt/*β*‐catenin signaling pathway has long been considered an attractive target for effective cancer therapy. Recently, several small molecules have been identified as the inhibitors of the Wnt/*β*‐catenin signaling pathway, such as the inhibitors of Wnt production (IWPs) and Wnt response (IWRs),^[^ ^32^ ^]^ ETC‐159,^[^ ^33^ ^]^ and JW55.^[^ ^34^ ^]^ However, using these small molecules for systemic cancer treatment still has several potential risks, including i) unsatisfactory pharmacokinetics and pharmacodynamics; and ii) potential toxicity owing to their nature of small chemical compounds.

LncRNAs are a class of newly identified noncoding transcripts that are widely involved in the intrinsic signaling networks of tumor cells.^[^ ^9^ ^]^ In recent years, series of radioresistance‐associated lncRNAs (e.g., HOTAIR, BOKAS, and lncRNA‐p21) have been reported and regulating their expression can induce multiple biological changes, such as blockage of DNA damage repair and promotion of apoptosis.^[^ ^12^, ^35^ ^]^ Genome‐wide analysis has shown that a large fraction of the human genome (≈98%) cannot encode proteins but can be transcribed into “non‐coding RNAs” that may exert their biological functions.^[^ ^13^ ^]^ Therefore, the number of currently identified radioresistance‐associated lncRNAs is still very low and much more efforts need to be made to discover new functional lncRNAs and elucidate their regulatory mechanisms. In this work, we examined the lncRNA expression profiles of locally recurrent tumors of TNBC patients who received postoperative radiotherapy, and identified lncAFAP1‐AS1 as a key factor in regulating radiosensitivity. After using large numbers of clinical samples to validate the association of lncAFAP1‐AS1 with radioresistance and extensively exploring its molecular mechanism, we demonstrated that this lncRNA can induce radioresistance via activating the Wnt/*β*‐catenin signaling pathway and its expression level in tumor tissues could be used to predict the outcome of TNBC radiotherapy.

Currently, numerous functional lncRNAs are known and their regulatory mechanisms have been also extensively elucidated. However, few efforts have been made to develop effective in vivo regulation strategies to improve therapeutic outcomes. RNA interference (RNAi) technology is the most convenient approach due to its strong ability to silence the expression of target gene(s) of interest, including those that encode “undruggable” proteins. However, due to the polyanionic and biomacromolecular characteristics of RNAi therapeutics, such as siRNAs, specific delivery vehicles are required to facilitate in vivo siRNA delivery.^[^ ^26^, ^36^ ^]^ Nanotechnology has shown great promise for improvement of in vivo siRNA delivery, and several RNAi NP platforms have been marketed (e.g., Onpattro and Givlaari) or entered into early phase clinical trials for the treatment of various diseases, especially liver diseases.^[^ ^27^ ^]^ However, effective and safe systemic delivery of siRNAs into tumor cells in vivo remains challenging, due to the complexities and heterogeneity of solid tumors.^[^ ^27^ ^]^

We had developed various polymeric NPs especially bioresponsive NPs for in vivo siRNA delivery and cancer therapy.^[^ ^15^, ^37^ ^]^ These bioresponsive NPs can respond to biological stimuli to achieve efficient intracellular siRNA delivery and better anticancer effect. Among them, reduction‐responsive NPs are particularly beneficial for systemic delivery of biomacromolecules that need to be released into the cytoplasm for therapeutic effects, because the GSH concentration in tumor cells is around 100–1000‐fold higher than that in the extracellular fluids.^[^ ^18^ ^]^ In this work, considering that tumor cells usually adaptively generate higher level of GSH under radiation to scavenge ROS and maintain intracellular redox homeostasis,^[^ ^6^ ^]^ we used reduction‐responsive PDSA NP platform for in vivo siAFAP1‐AS1 delivery and investigated its clinical translation potential for reversal of radioresistance and effective cancer radiotherapy. In the xenograft and metastatic radioresistant tumor models, this reduction‐responsive RNAi NP platform can indeed reverse radioresistance and shows the following features: i) long blood circulation and high tumor accumulation; ii) GSH‐triggered fast intracellular siAFAP1‐AS1 release for efficient lncAFAP1‐AS1 silencing and subsequent blockage of the Wnt/*β*‐catenin signaling pathway; iii) scavenging the elevated GSH under radiation by PDSA polymer to disrupt intracellular redox homeostasis, which synergizes with lncAFAP1‐AS1 silencing for combinational reversal of radioresistance to enhance cancer radiotherapy effect.

## 4 Conclusion

In conclusion, we have identified and revealed the important biological function of lncAFAP1‐AS1 in radioresistant TNBC. This lncRNA can activate the Wnt/*β*‐catenin signaling pathway and thus induce radioresistance of TNBC via promoting cell proliferation, migration, and invasion. Using reduction‐responsive PDSA NPs to transport siAFAP1‐AS1 can efficiently silence lncAFAP1‐AS1 expression and currently scavenge the elevated GSH, leading to synergistic reversal of radioresistance. Systemic delivery of siAFAP1‐AS1 with the reduction‐responsive NPs can synergistically enhance the radiosensitivity and significantly improve the radiotherapy effect in both xenograft and metastatic tumor models. The new RNAi NP platform developed in this work shows a great potential for the treatment of TNBC patients with recurrence and metastasis.

## 5 Experimental Section

##### Patients and Tissue Samples

TNBC samples of 125 female patients between September 2005 and May 2017 were collected from the Breast Tumor Center, Sun Yat‐sen Memorial Hospital, Sun Yat‐sen University. Among these 125 patients, 44 patients received postoperative radiotherapy and 22 patients had local recurrence and metastasis. The tumor samples included 125 surgical resected tumors and two core needle biopsies of locally recurrent tumors of patients receiving postoperative radiotherapy. Among these collected tumor samples, matched tumor tissues collected from two patients before radiotherapy (surgically resected tumors) and after postoperative radiotherapy with local recurrence and metastasis (core needle biopsies). Pathological diagnosis, Ki67, and other biomarkers were verified independently by two pathologists. Radioresistant patients were defined as those with local recurrence at the breast and/or lymph nodes after completion of radiotherapy. All human samples were collected with informed consents from the donors according to the International Ethical Guidelines for Biomedical Research Involving Human Subjects (CIOMS). The study was approved by the Institutional Review Board (IRB) of Sun Yat‐sen Memorial Hospital.

##### Cell Culture and Antibodies

MDA‐MB‐231 cells were obtained from American Type Culture Collection (ATCC) and cultured in Dulbecco's Modified Eagle Medium (DMEM) with 10% fetal bovine serum (FBS) at 37 °C in a humidified atmosphere containing 5% CO_2_. The radioresistant cells (MDA‐MB‐231R) were established by giving X‐ray radiation to MDA‐MB‐231 cells three times per week at a dose of 2 Gy. Cells with a death rate less than 5% under radiation were determined as radioresistant cells using the linear quadratic model and single‐hit multitarget model. The antibodies were purchased from Cell Signaling Technology and the detailed information is as follows: GAPDH rabbit mAb (D16H11, #8884), total *β*‐catenin rabbit mAb (D10A8, #8480), active *β*‐catenin rabbit mAb (D2A8Y, #19 087), GSK‐3*β* rabbit mAb (D5C5Z, #12 456), p‐GSK‐3*β* rabbit mAb (5B3, #9323), CK1 rabbit mAb (#2566), Ki‐67 mouse mAb (8D5, #9449), histone H2A.X rabbit mAb (D17A3, #7631), cleaved caspase‐3 rabbit mAb (A1E, #9664), digoxigenin (DIG) rabbit mAb (D8Q9J, #14 682), antirabbit IgG horseradish peroxidase (HRP)‐linked secondary Ab (#7074).

##### RNA Preparation and Microarray Analysis

Total RNA in the cells and tumor samples (surgically resected tumors of two TNBC patients before radiotherapy and locally recurrent tumors of the same patients receiving postoperative radiotherapy) was extracted using Trizol (Invitrogen, USA) according to the manufacturer's instructions. Agilent human lncRNA Microarray V6 (4 × 180 K) was used to analyze the global profiling of human lncRNAs and protein‐coding transcripts in the samples. The raw data were extracted by Feature Extraction software and further quantile normalized and exhibited as log2 transform using the GeneSpring software. The intensity was used to generate the heatmap by MeV4.7.

##### Online Dataset

The correlation of lncAFAP1‐AS1 expression in TNBC patients with the survival outcome from TCGA database was downloaded from broad Dashboard‐stddata (https://confluence.broadinstitute.org/display/GDAC/Dashboard-Stddata). Kaplan–Meier curve and log‐rank test were used to compare OS and DFS in different patient groups.

##### ISH and Data Analysis

LncAFAP1‐AS1 expression in paraffin‐embedded tumor samples was examined using an ISH optimization kit (Roche, Switzerland) according to the manufacturer's instructions. In brief, the tumor slides were treated with pepsin for 10 min at room temperature and incubated with 500 × 10^−9^ m of digoxigenin (DIG)‐labeled probe targeting lncAFAP1‐AS1 (RiboBio, China) at 55 °C for 4 h. After washing with PBS containing 0.1% Tween 20 (PBST) (3 × 5 min) and blocking with 10% FBS for 30 min, the slices were incubated with secondary antidigoxigenin (anti‐DIG) antibody at 4 °C overnight. Subsequently, the slides were washed with PBST (3 × 5 min) and incubated with the antirabbit IgG HRP‐linked antibody for 1 h. After adding diaminobenzidine and hematoxylin, the slides were finally viewed under an optical microscope. The lncAFAP1‐AS1 expression was analyzed by combining the percentage of positively‐stained tumor cells and staining intensity of positively‐stained tumor cells. The staining intensity was graded as follows: 0, no staining; 1, weak staining (light purple); 2, moderate staining (purple‐dark purple); 3, strong staining (dark purple). The percentage of cells at each staining intensity level was calculated and an H‐score was finally assigned using the following formula: [1 × (% cells 1+) + 2 × (% cells 2+) + 3 × (% cells 3+)]. The median value was used as the cut‐off to define high lncAFAP1‐AS1 expression and low lncAFAP1‐AS1 expression in the tumor samples collected from 125 TNBC patients.

##### qRT‐PCR

Total RNA was extracted from the cultured cells using Trizol and 1 µg of RNA was then reverse transcribed into cDNAs using a Superscript First‐Strand cDNA Synthesis Kit (18080‐051, Invitrogen, USA). qRT‐PCR analysis was performed using SYBR Premix Ex Taq II kit (DRR081A, TAKARA, Japan) on a LightCycler 480 System (Roche, Switzerland).

##### FISH

The cultured cells were fixed with 4% paraformaldehyde, permeabilized in 0.5% Triton X‐100 on ice for 1 h, and then hybridized with the DIG‐labeled probe targeting lncAFAP1‐AS1 at room temperature overnight. After incubating with Cy3‐conjugated anti‐DIG antibody at 4 °C overnight, the nuclei were stained with Hoechst 33 342 and the cells were viewed under confocal laser scanning microscope (CLSM) (A1, Nikon, Japan).

##### RNA Pulldown Assay

The 3′‐end biotin‐labeled lncAFAP1‐AS1 was first transcribed using T7 High Yield Transcription Kit (AM1334, Ambion, USA) and then purified with MEGAclear Kit (AM1908, Ambion, USA) according to the manufacturer's instructions. Subsequently, 5 pmol of purified RNA was withdrawn and heated to 95 °C for 5 min and then placed at room temperature to allow the formation of proper secondary structure. This folded RNA was then incubated with the lysates of the cultured cells treated with lysis buffer supplemented with anti‐RNase and protease/phosphatase inhibitor cocktail. 1 h later, Dynabeads Streptavidin magnetic beads (65801D, Invitrogen, USA) were added and the mixture was allowed to shake at room temperature for 5 min. Subsequently, the magnetic beads were isolated and washed with lysis buffer (3 × 5 min). For the silver staining, the specific RNA‐protein binding complexes on the magnetic beads were running with the sodium dodecyl sulfate‐polyacrylamide gel electrophoresis (SDS‐PAGE) gel. Subsequently, the gel was fixed in fixation solution (40% ethanol, 10% acetic acid, 50% water) for 30 min. After rinsed with 0.5% dichromate for 5 min, the gel was equilibrated with 0.1% silver nitrate for 30 min. The specific RNA‐binding proteins were finally collected for mass spectrometry. For the Western blot analysis, the RNA‐protein binding complexes on the magnetic beads were added to the SDS‐PAGE gel and separated by gel electrophoresis according to the protocol described below.

##### Western Blot

Equal amounts of proteins, as determined with a bicinchoninic acid protein assay kit (Pierce/Thermo Scientific) according to the manufacturer's instructions, were added to the SDS‐PAGE gel and separated by gel electrophoresis. After transferring the proteins from gel to polyvinylidene difluoride membrane, the blots were blocked with 5% nonfat milk in PBST at room temperature for 1 h and then incubated with different antibodies. The protein expression was detected with the antirabbit IgG HRP‐linked secondary antibody and an enhanced chemiluminescence detection system.

##### RIP

The RIP assay was performed using the Magna RIP RNA‐Binding Protein Immunoprecipitation Kit (17‐700, Millipore, USA) according to the manufacturer's instructions. In brief, lysates of the cultured cells were incubated with magnetic beads with 5 µg of IP‐grade antibody and incubated in IP buffer at 4 °C overnight. Subsequently, the RNA was collected, purified, and finally quantified by qRT‐PCR. Input control and normal antirabbit IgG control were also tested to ensure the accuracy of detected signals from the protein‐bound RNA.

##### Preparation and Characterizations of NPs

The classic nanoprecipitation method was used to prepare the siRNA loaded NPs.^[^ ^38^ ^]^ In brief, a mixture of 1 nmol siRNA (0.1 nmol µL^−1^ aqueous solution) and 50 µL of G0‐C14 (5 mg mL^−1^ in dimethylformamide) in an N/P molar ratio of 1:20 was prepared, which was then added to the mixture of 200 µL of PDSA polymer solution (20 mg mL^−1^ in dimethylformamide) and 140 µL of DSPE‐PEG_3k_ solution (20 mg mL^−1^ in dimethylformamide). Under vigorous stirring (1000 rpm), the mixture was added dropwise to 5 mL of deionized water. The formed NP dispersion was transferred to an ultrafiltration device (EMD Millipore, MWCO 100 K) and centrifuged to remove the organic solvent and free compounds. After washing with deionized water, the obtained NPs were collected and dispersed in PBS solution at a siRNA concentration of 1 nmol mL^−1^. Dynamic light scattering (Brookhaven, USA) was used to examine the size and zeta potential of the siRNA NPs. The NP morphology was viewed on a Tecnai G^2^ Spirit BioTWIN transmission electron microscope (FEI, USA). To determine siRNA encapsulation efficiency (EE%), Cy5‐labeled siRNA was encapsulated into the NPs according to the method described above. A small volume (5 µL) of the NP solution was taken to mix with 20‐fold dimethyl sulfoxide. The fluorescence intensity of Cy5‐labeled siRNA was measured using a Synergy HT multimode microplate reader (BioTek, USA). The amount of loaded siRNA in the NPs was calculated according to the standard curve.

##### LncAFAP1‐AS1 Silencing and Overexpression

MDA‐MB‐231R cells were seeded in 6‐well plates (50 000 cells per well) and incubated in 2 mL of DMEM containing 10% FBS for 24 h. Subsequently, the medium was replaced by fresh medium and then the NPs(si‐1), Lipo2k/si‐1, or Lipo2k/si‐2 complexes were added to silence the lncAFAP1‐AS1 expression. After incubation for 24 h, the cells were washed with PBS solution and further incubated in fresh medium for another 48 h. For the lncAFAP1‐AS1 overexpression experiment, parental MDA‐MB‐231 cells were seeded in 6‐well plates (50 000 cells per well) and then incubated with the Lipo2k/plasmid complexes for 24 h. After washing with PBS solution, the cells were further incubated in fresh medium for another 48 h. After verifying the successful lncAFAP1‐AS1 silencing or overexpression by qRT‐PCR, the cells were used for flow cytometry analysis, migration and invasion assay, and Western blot analysis.

##### Flow Cytometry

MDA‐MB‐231R or parental MDA‐MB‐231 cells were seeded in 6‐well plates (50 000 cells per well) and incubated in 2 mL of DMEM containing 10% FBS for 24 h. After replacing the medium, lncAFAP1‐AS1 was silenced in MDA‐MB‐231R cells or overexpressed in parental MDA‐MB‐231 cells. Subsequently, various doses of X‐ray radiation were applied, and the cells were finally trypsinized and stained with Annexin IV (20 µg mL^−1^) and propidium iodide (50 µg mL^−1^) for flow cytometry analysis using a Cytomics FC 500 instrument (Beckman, USA).

##### Cell Migration and Invasion

Migration assay was performed using 24‐well Boyden chamber (Corning, USA) with 8 M‐insert while invasion assay was carried out using the same device coated with 20% Matrigel (Corning, USA). Various doses of X‐ray radiation were first applied to MDA‐MB‐231R with lncAFAP1‐AS1 silencing or parental MDA‐MB‐231 cells with lncAFAP1‐AS1 overexpression. Subsequently, the cells were trypsinized and transferred to the upper chamber containing DMEM without FBS (1000 cells per well). 24 h later, cells attached to the bottom of the upper chamber were counted and imaged to show the cell migration and invasion profiles.

##### Animals

BALB/c normal mice and nude mice (female, 4–5 weeks old) were purchased from the Sun Yat‐sen University Experimental Animal Center (Guangzhou, China). All in vivo studies were performed in accordance with a protocol approved by the Institutional Animal Care and Use Committee at Sun Yat‐sen University.

##### Xenograft and Metastatic Tumor Model

MDA‐MB‐231R xenograft tumor model was constructed by subcutaneous injection with 200 µL of MDA‐MB‐231R cell suspension (a mixture of DMEM medium and Matrigel in 1:1 volume ratio) with a density 1 × 10^7^ cells mL^−1^ into the back region of healthy nude mice. When the tumor volume reached around 150 mm^3^, the mice were used for the in vivo experiments. For the establishment of the metastatic tumor model, Luc‐expressing MDA‐MB‐231R cells (5 × 10^6^) were intravenously injected into the healthy nude mice and tumor growth was monitored by examining the average radiance of the tumor sites using bioluminescence imaging. Prior to imaging, D‐luciferin substrate was intraperitoneally injected to mice at a dose of 150 mg kg^−1^ and the mice were viewed using an IVIS Lumina III (PerkinElmer, USA) imaging system.

##### Inhibition of Xenograft Tumor Growth

MDA‐MB‐231R xenograft tumor‐bearing mice were randomly divided into six groups (*n* = 5) and received the treatment with i) PBS, ii) PBS followed ionizing radiation, iii) NPs(siCTL), iv) NPs(siCTL) followed by ionizing radiation, v) NPs(si‐1), and vi) NPs(si‐1) followed by ionizing radiation. The siRNA loaded NPs were intravenously injected into the mice once every 2 days at a 1 nmol siRNA dose per mouse. After three rounds of treatments, 10 Gy of ionizing radiation was applied twice, once at 24 h and once at 8 days post the final injection. The tumor growth was monitored every 2 days by measuring perpendicular diameters using a caliper and tumor volume was calculated as follows

(1) $$V=W^{2}\times L/2$$

where *W* and *L* are the shortest and longest diameters, respectively.

##### Inhibition of Metastatic Tumor Growth

MDA‐MB‐231R metastatic tumor‐bearing mice were randomly divided into five groups (*n* = 3) and received the treatment with i) PBS, ii) NPs(siCTL), iii) NPs(siCTL) followed by ionizing radiation, iv) NPs(si‐1), and v) NPs(si‐1) followed by ionizing radiation. The siRNA loaded NPs were intravenously injected into the mice once every 2 days at a 1 nmol siRNA dose per mouse. After three rounds of treatments, 10 Gy of ionizing radiation was applied twice, once at 24 h and once at 8 days post the final injection. The tumor burden was monitored at day 0, 7, and 14 using bioluminescence imaging system according to method described above.

##### Statistical Analysis

The in vitro data were presented as mean ± S.D. of three independent experiments. All statistical analyses were performed using SPSS 16.0 statistical software package and Graphpad Prism 8. Unpaired two‐sided Student's *t*‐test and one‐way ANOVA was used to compare cell viability, colony formation, apoptosis, and tumor volume with different treatments, and post hoc tests were used to test difference between groups. Kaplan–Meier curves and log‐rank test were used to compare OS and DFS in different patient groups. In all cases, *p < 0.05, **p < 0.01, and ***p < 0.001.

## Conflict of Interest

The authors declare no conflict of interest.

## Supporting information

Supporting Information
