# Supplementary material for: Nanoparticles (NPs)‐Meditated LncRNA AFAP1‐AS1 Silencing to Block Wnt/β‐Catenin Signaling Pathway for Synergistic Reversal of Radioresistance and Effective Cancer Radiotherapy
Source: Adv Sci (Weinh). 2020 Aug 5;7(18):2000915. doi: 10.1002/advs.202000915 (PMC7509644; doi:10.1002/advs.202000915)
Supplement: Supplementary file 1 — Supporting Information [file ADVS-7-2000915-s001.pdf]

2020 Wiley-VCH GmbH

ADVANCED SCIENCE
Open Access

Supporting Information

for *Adv. Sci.,* DOI: 10.1002/advs.202000915

Nanoparticles (NPs)-Meditated LncRNA AFAP1-AS1 Silencing to Block Wnt/*β*-Catenin Signaling Pathway for Synergistic Reversal of Radioresistance and Effective Cancer Radiotherapy

Zhuofei Bi, Qingjian Li, Xiaoxiao Dinglin, Ying Xu, Kaiyun You, Huangming Hong, Qian Hu, Wei Zhang, Chenchen Li, Yujie Tan, Ning Xie, Wei Ren, Chuping Li, Yimin Liu, Hai Hu, Xiaoding Xu, and Herui Yao**

Supplementary Material

of

Nanoparticles (NPs)-Meditated LncRNA AFAP1-AS1 Silencing to Block Wnt/β-Catenin Signaling Pathway for Synergistic Reversal of Radioresistance and Effective Cancer Radiotherapy

Zhuofei Bi[1,2,3], Qingjian Li[3], Xiaoxiao Dinglin[3,4], Ying Xu[1,2], Kai-yun You[3], Huangming Hong[3], Qian Hu[3], Wei Zhang[3], Chenchen Li[3], Yujie Tan[3], Ning Xie[3], Wei Ren[3], Chuping Li[3], Yimin Liu[3] Hai Hu[1,2,3], Xiaoding Xu[1,2*], Herui Yao[1,2,3,4*]

[1]Guangdong Provincial Key Laboratory of Malignant Tumor Epigenetics and Gene Regulation, Medical Research Center, Sun Yat-sen Memorial Hospital, Sun Yat-sen University,

Guangzhou, China.

[2]RNA Biomedical Institute, Sun Yat-sen Memorial Hospital, Sun Yat-sen University, Guangzhou, China.

[3]Department of Oncology, Sun Yat-sen Memorial Hospital, Sun Yat-sen University, Guangzhou, China.

[4]Breast Tumor Center, Sun Yat-sen Memorial Hospital, Sun Yat-sen University, Guangzhou, China.

*Corresponding author: yaoherui@mail.sysu.edu.cn; xuxiaod5@mail.sysu.edu.cn

1. Materials

1,2-Distearoyl-*sn*-glycero-3-phosphoethanolamine-*N*-[methoxy(polyethylene glycol)-3000] ($DSPE-PEG_{3k}$) was purchased from Avanti Polar Lipids and used directly. L-Cystine dimethyl ester dihydrochloride ($(H-Cys-OMe)_2.2HCl$), reductive glutathione (GSH), dimethyl sulfoxide (DMSO), dimethylformamide (DMF), and triethylamine were purchased from Sigma-Aldrich and used as received. The reduction-responsive poly(disulfide amide) (PDSA) polymer was synthesized via one-step polycondensation of $(H-Cys-OMe)_2.2HCl$ and sebacoyl chloride according to our previous study [1]. The chemical structure and molecular weight of the PDSA polymer (M_w = 7800) were respectively examined by proton nuclear magnetic resonance (^{1}HNMR) and gel permeation chromatography (GPC). Cationic lipid-like compound alkyl-modified polyamidoamine (PAMAM) dendrimer (G0-C14) was synthesized through ring opening of 1,2-epoxytetradecane by generation 0 of (PAMAM) dendrimer according to previous report [2]. Poly(lactic-*co*-glycolic acid) (PLGA, 0.1-0.25 dL/g) was purchased from LACTEL Absorbable Polymers and used directly. LncAFAP1-AS1 siRNA (siAFAP1-AS1), fluorescent dye Cy5-labeled lncAFAP1-AS1 siRNA, and luciferase siRNA (siLuc) were acquired from RiboBio Co., Ltd (Guangzhou, China). The siAFAP1-AS1 sequences are as follows: sequence 1 (si-1), 5'-GCA CAG GUU CUC CAA ACA ATT-3' (sense), 5'-UUG UUU GGA GAA CCU GUG CTT-3' (antisense); sequence 2 (si-2), 5'-GCU ACU UCU GUC UCA UUA ATT-3' (sense), 5'-UUA AUG AGA CAG AAG UAG CTT-3' (antisense). Cy5 was labeled at the 5'-end of both the sense and antisense strands of si-1. The siLuc sequences are as follows: siLuc, 5'-CUU ACG CUG AGU ACU UCG AdTdT-3' (sense) and 5'-UCG AAG UAC UCA GCG UAA GdTdT-3' (antisense). The primers for qRT-PCR analysis are as follows: lncAFAP1-AS1, 5'-AGT TTG GGG TAT GTT TTT ATT AGT A-3' (forward sequence), 5'-AAA ATA ACA CTT CCC CTC CCA ACC T-3' (reverse sequence); GAPDH, 5'-ATC ACC ATC TTC CAG GAG CGA-3' (forward sequence), 5'-CCT TCT CCA TGG TGG TGA AGA C-3' (reverse sequence). Dulbecco's Modified Eagle's

Medium (DMEM), penicillinestreptomycin, trypsin, and fetal bovine serum (FBS) were purchased from Invitrogen Corp. and used received. All other reagents and solvents are of analytical grade and used without further purification.

2. Screening of reduction-responsive NPs

The siLuc loaded NPs were prepared using the PDSA polymers with different chemical structure and molecular weight. Subsequently, Luc-expressing HeLa cells were seeded in 96-well plates (5,000 cells per well) and incubated in 0.1 mL of RPMI 1640 medium) with 10% FBS for 24 h. Thereafter, the medium was replaced by fresh medium and siLuc loaded NPs were added. After 24 h incubation, the cells were washed with PBS buffer and allowed to incubate in fresh medium for another 48 h. The Luc expression was determined using Steady-Glo luciferase assay kits. Cytotoxicity was measured using AlamarBlue assay according to the manufacturer's protocol. The luminescence or fluorescence intensity was measured using a microplate reader, and the average value of five independent experiments was collected. According to the above experiments, the PDSA8a nanoplatform with the best gene silencing efficacy was selected for the siAFAP1-AS1 delivery.

3. Evaluation of the reduction response of the siRNA loaded NPs

The si-1 loaded PDSA8a NPs (denoted NPs(si-1)) were prepared and then dispersed in 1 mL of PBS containing GSH with different concentrations. At a predetermined interval, the size of the NPs(si-1) was examined by dynamic light scattering (DLS), and the morphology of the NPs(si-1) was observed by transmission electron microscope (TEM). To examine the influence of reduction response on the siRNA release from the NPs, Cy5-labeled si-1 was encapsulated into the NPs (denoted NPs(Cy5-si-1)). Subsequently, the NPs were dispersed in 1 mL of PBS (pH 7.4) and then transferred to a Float-a-lyzer G2 dialysis device (MWCO 100 kDa, Spectrum) that was immersed in PBS buffer containing GSH at 37 ^{o}C. At a

predetermined interval, 5 µL of the NP solution was withdrawn and mixed with 20-fold DMSO. The fluorescence intensity of Cy5-labeled si-1 was determined using a microplate reader.

4. Pharmacokinetics study

Healthy male BALB/c female mice were randomly divided into two groups (n = 3) and given an intravenous injection of either (i) NPs(Cy5-si-1) or (ii) naked Cy5-si-1 at a 1 nmol siRNA dose per mouse. At predetermined time intervals, orbital vein blood (20 µL) was withdrawn using a tube containing heparin, and the wound was pressed for several seconds to stop the bleeding. The fluorescence intensity of Cy5-labeled siRNA in the blood was determined by microplate reader. The blood circulation half-life ($t_{1/2}$) was calculated according to previous report [3].

5. Biodistribution

MDA-MB-231R xenograft tumor-bearing female nude mice were randomly divided into two groups (n = 3) and given an intravenous injection of either (i) NPs(Cy5-si-1) or (ii) naked Cy5-si-1 at a 1 nmol siRNA dose per mouse. Twenty-four hours after the injection, the mice were imaged using an IVIS Lumina Ⅲ imaging system. Organs and tumors were then harvested and imaged. To quantify the accumulation of NPs in tumors and organs, the fluorescence intensity of each tissue was quantified by Image-J.

6. Immune response

Healthy female BALB/c mice were randomly divided into six groups (n = 3) and given an intravenous injection of either (i) PBS, (ii) naked siCTL, (iii) naked si-1, (iv) PDSA NPs, (v) NPs(siCTL) or (vi) NPs(si-1) at a 1 nmol siRNA dose per mouse. Twenty-four hours after injection, blood was collected and serum isolated for representative cytokine analysis by

enzyme-linked immunosorbent assay or ELISA (PBL Biomedical Laboratories and BD Biosciences) according to the manufacturer's instructions.

7. Blood and histological analysis

Healthy female BALB/c mice were randomly divided into six groups (n = 3) and given an intravenous injection of either (i) PBS, (ii) naked siCTL, (iii) naked si-1, (iv) PDSA NPs, (v) NPs(siCTL) or (vi) NPs(si-1) at a 1 nmol siRNA dose per mouse. After three consecutive injections, blood was collected at 24 h post the final injection and serum isolated for routine blood analysis. And the main organs were collected, fixed with 4% paraformaldehyde, and embedded in paraffin. Tissue sections were stained with hematoxylin-eosin (H&E) and then viewed under an optical microscope.

8. Immunohistochemistry (IHC) staining

IHC staining was performed on formalin-fixed paraffin-embedded tumor sections. Briefly, tumor slides were first heated to 60 ^{o}C for 1 h, desparaffinized with xylene (3 × 5 min), and washed with different concentrations of alcohol. After retrieval of antigen using DAKO target retrieval solution at 95-99 ^{o}C for 40 min, followed by washing, the slides were blocked with peroxidase blocking buffer (DAKO Company) for 5 min. After washing DAKO buffer the slides were incubated with PLK1 rabbit antibody (Abcam) diluted in DAKO antibody solution for 1 h. The slides were then washed and incubated with peroxidase-labeled polymer for 30 min. After washing and staining with DAB+ substrate-chromogen solution and hematoxylin, the slides we remounted and viewed under a MVX10 MacroView Dissecting scope equipped with OlympusDP80 camera.

References

[1] Wu J, *et al.* (2015) Hydrophobic cysteine poly(disulfide)-based redox-hypersensitive

nanoparticle platform for cancer theranostics. *Angew Chem Int Ed Engl* 54(32):9218-9223.

[2] Xu X, *et al*. (2013) Enhancing tumor cell response to chemotherapy through nanoparticle-mediated codelivery of siRNA and cisplatin prodrug. *Proc Natl Acad Sci USA* 110(46):18638-18643.

[3] Winter H, *et al.* (2013) Effect of a high-calorie, high-fat meal on the bioavailability and pharmacokinetics of PA-824 in healthy adult subjects. *Antimicrob Agents Chemother* 57(11): 5516-5520.

Table S1. The information of tumor tissues from recurrent TNBC patients

Patient	Age	Subtype	Positive Node	Radiation Model	Dose	Fraction	Metastasis site
NO.1	67	TNBC	5	IMRT	66	33	Chest wall
NO.2	53	TNBC	0	IMRT	60	20	Lymph node

TNBC (Triple negative breast cancer); IMRT (Intensity-Modulated Radiotherapy)

Table S2. Correlation of lncAFAP1-AS1 expression with clinicopathological status in 125 TNBC patients

	AFAP1-AS1 score		
Variable	H-Score ≤ 150 (61)	H-Score > 150 (64)	*P* value
Age			
≤ 35	11	8	0.495
> 35	50	56	
Grade			
I+II	28	35	0.373
III	33	29	
T stage			
T1+T2	55	58	0.584
T3+T4	6	6	
N stage			
N0	46	36	0.038
N1+N2+N3	15	18	
M stage			
M0	59	58	0.274
M1	2	6	
TNM stage			
I+II	53	45	0.030
III+IV	8	19	
Ki67 expression			
≤ 14%	4	7	0.531
> 14%	57	57	

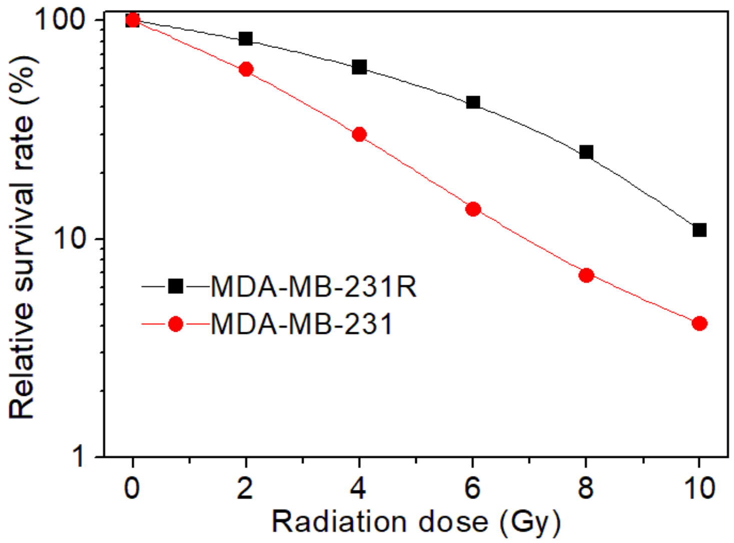

Figure S1. Relative survival rate of MDA-MB-231R cells and parental MDA-MB-231 cells treated with different doses of X-ray radiation.

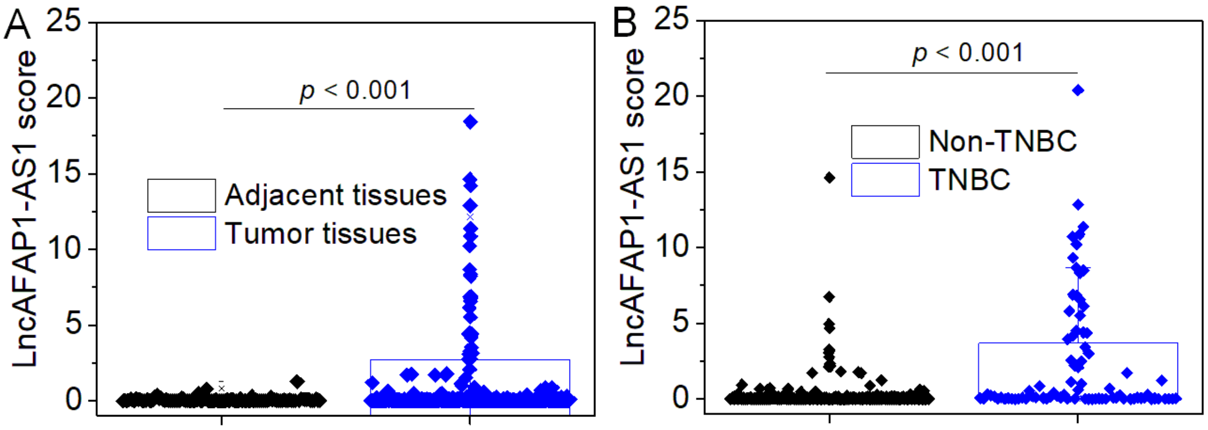

Figure S2. TCGA database showing the lncAFAP1-AS1 score in the adjacent (n = 105) and tumor tissues (n= 400) of breast cancer patients, and in the tumor tissues of TNBC (n = 84) and non-TNBC (n = 644) patients.

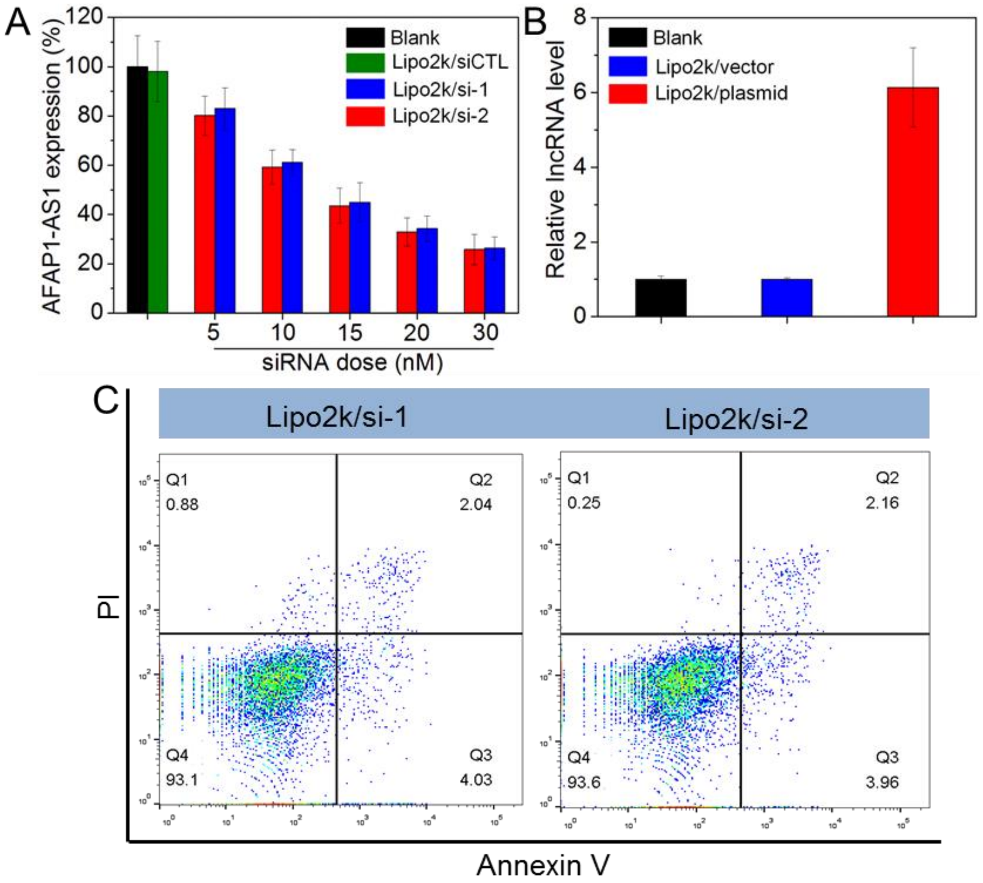

Figure S3. (A, B) qRT-PCR analysis of the lncAFAP1-AS1 expression in MDA-MB-231R cells treated with the Lipo2k/siCTL, Lipo2k/si-1, or Lopi2k/si-2 complexes at different siRNA doses (A), and in parental MDA-MB-231 cells treated with the Lipo2k/vector, or Lopi2k/plasmid complexes at a plasmid concentration of 1.5 μg/mL (B). (C) Flow cytometry profile of MDA-MB-231R cells treated with the Lipo2k/si-1 or Lipo2k/si-2 complexes at a siRNA dose of 15 nM.

No.	Protein	M_W (kDa)	Antisense	Sense
1	Catenin beta-1 [OS=Homo sapiens]	85.5	0	13
2	Heat shock cognate 71 kDa protein	70.9	2	20
3	Isoform 2 of Polyadenylate-binding protein 4	69.5	1	23
4	Methylcrotonoyl-CoA carboxylase subunit alpha,	80.4	1	13
5	Fragile X mental retardation syndrome-related protein 1	69.7	6	20
6	Heterogeneous nuclear ribonucleoprotein M	77.5	3	22
7	zinc finger CCCH-type antiviral protein 1	101.4	1	16
8	probable ATP-dependent RNA helicase DDX5	69.1	4	17
9	Insulin-like growth factor 2 mRNA-binding protein 2	66.1	0	11
10	Probable ATP-dependent RNA helicase DDX17	80.2	3	16

Figure S4. Top 10 proteins enriched in the lncAFAP1-AS1 pulldown complexes detected by mass spectrometry (MS).

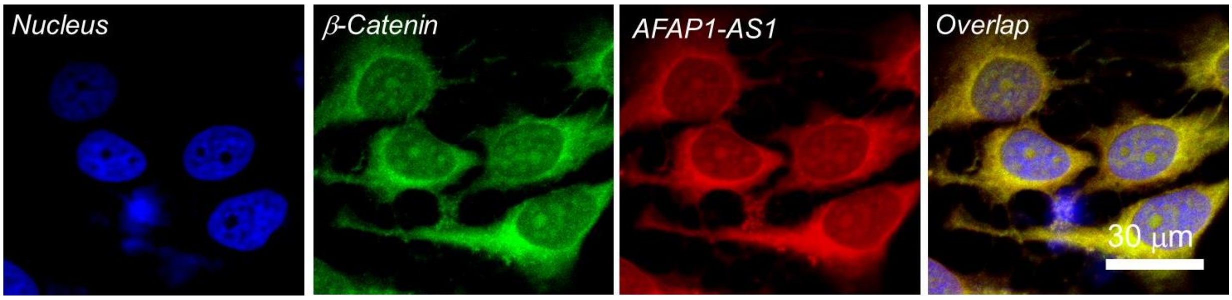

Figure S5. Fluorescence images of MDA-MB-231R cells observed under confocal laser scanning microscope (CLSM). The nucleus, β-catenin, and lncAFAP1-AS1 were stained with blue, green, and red fluorescence, respectively.

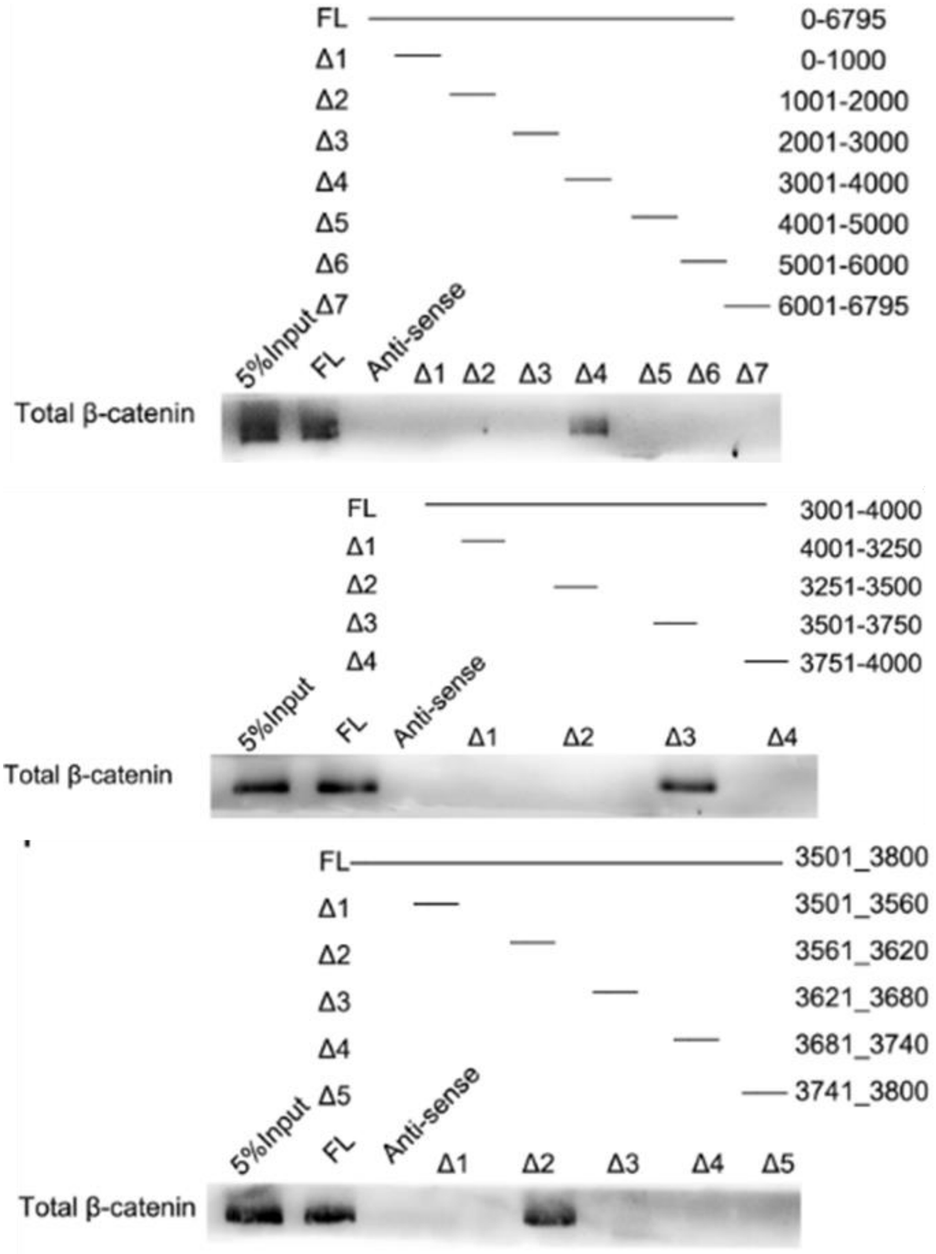

Figure S6. RNA pulldown assay followed by western blot analysis of the site of lncAPAP1-AS binding to β-catenin using **t**runcated lncAFAP1-AS1 containing 1-60 nt with the possible ability to bind β-catenin.

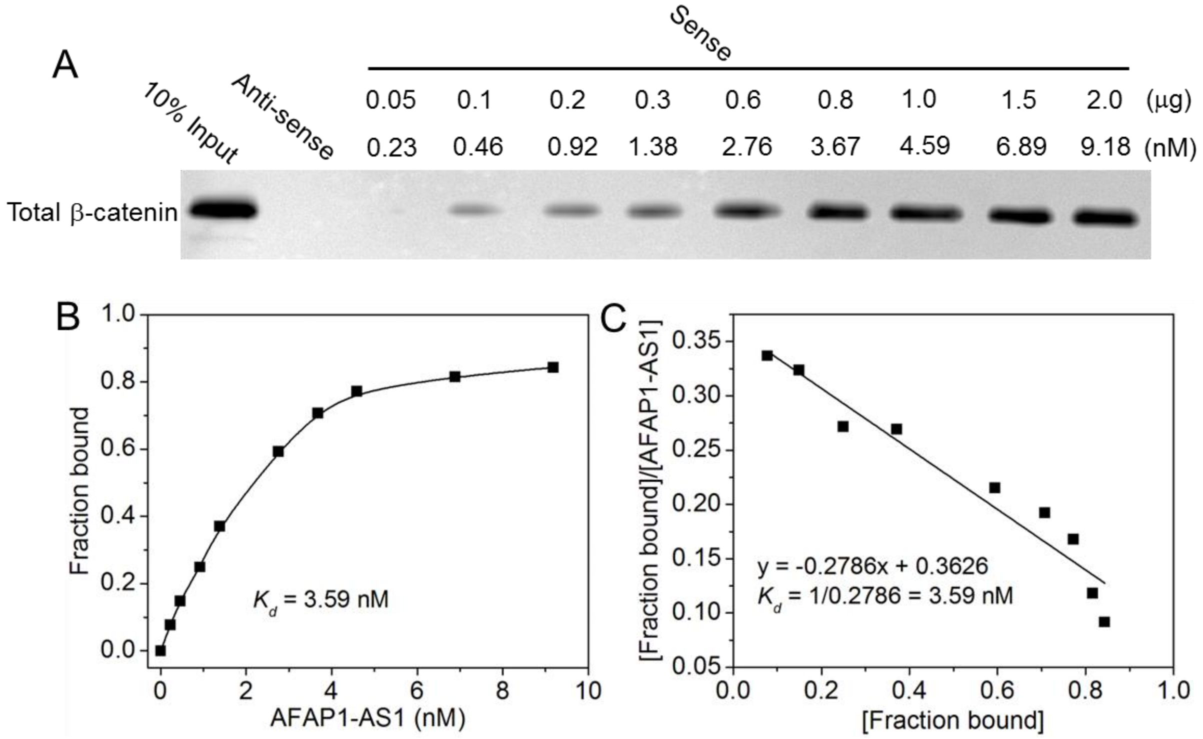

Figure S7. (A) Western blot analysis of the RNA-protein binding complexes after RNA pulldown of lncAFAP1-AS1 in MDA-MB-231R cells. (B) Dependence of β-catenin abundance upon the lncAFAP1-AS1 concentration. (C) Linear regression analysis of the data shown in (B) using the Scatchard equation.

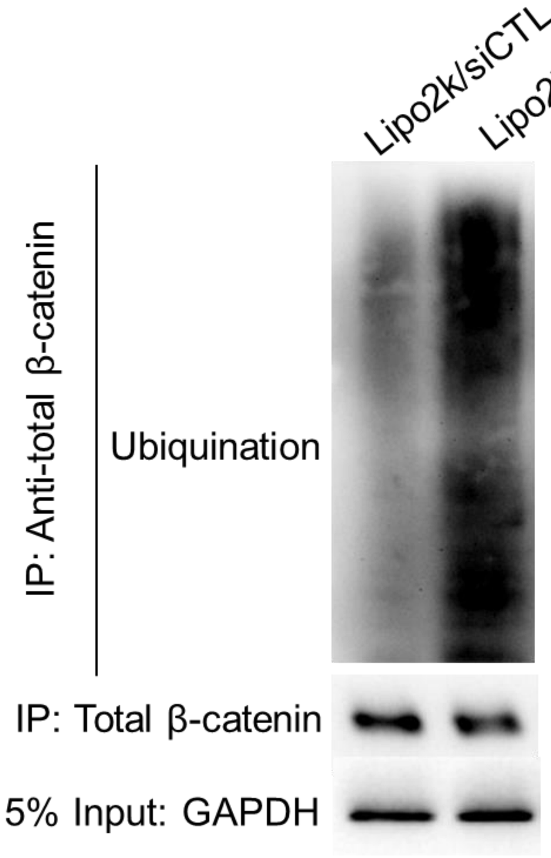

Figure S8. Western blot analysis of the ubiquitination of β-catenin in MDA-MB-231R cells treated with the Lipo2k/siCTL or Lipo2k/siAFAP1-AS1 complexes at a siRNA concentration of 30 nM. The cells were pre-treated with proteasome inhibitor MG132 (20 nM) for 4 h before collecting lysate.

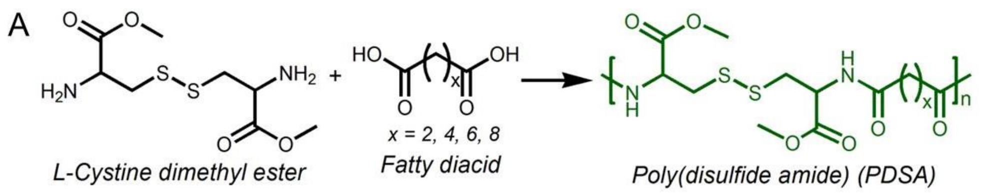

B

No.	PDSA2	PDSA4	PDSA6	PDSA8	PDSA8a	PDSA8b	PDSA8c
M_w (kDa)	3.1	4.3	5.7	7.3	7.8	15.2	16.6
Size (nm)	232.5	155.7	134.5	118.9	102.9	99.4	93.4
EE%	18.6	29.7	35.1	46.3	55.9	79.4	88.2
ξ (mV)	-1.16	-6.77	-8.12	-9.52	-11.21	-16.13	-20.12

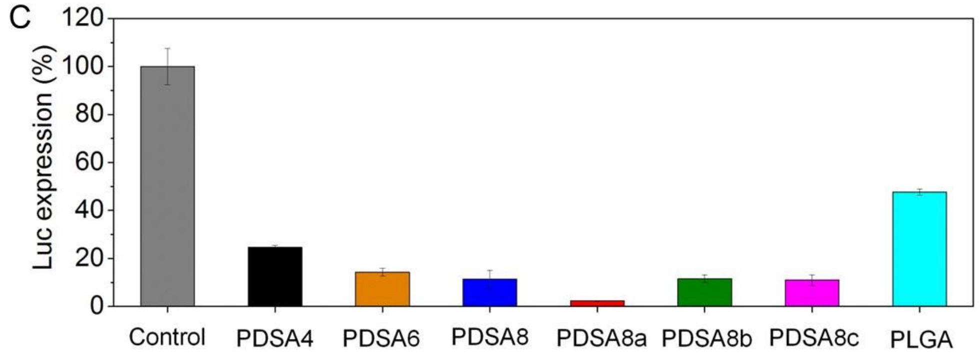

Figure S9. (A) Synthesis route of different PDSA polymers. (B) Molecular weight of the PDSA polymers, and the size, siRNA encapsulation efficiency (EE%), and zeta potential (ζ) of the siLuc loaded NPs made with the PDSA polymers. (C) Luc expression in the Luc-expressing HeLa cells treated with the siLuc loaded PDSA or PLGA NPs.

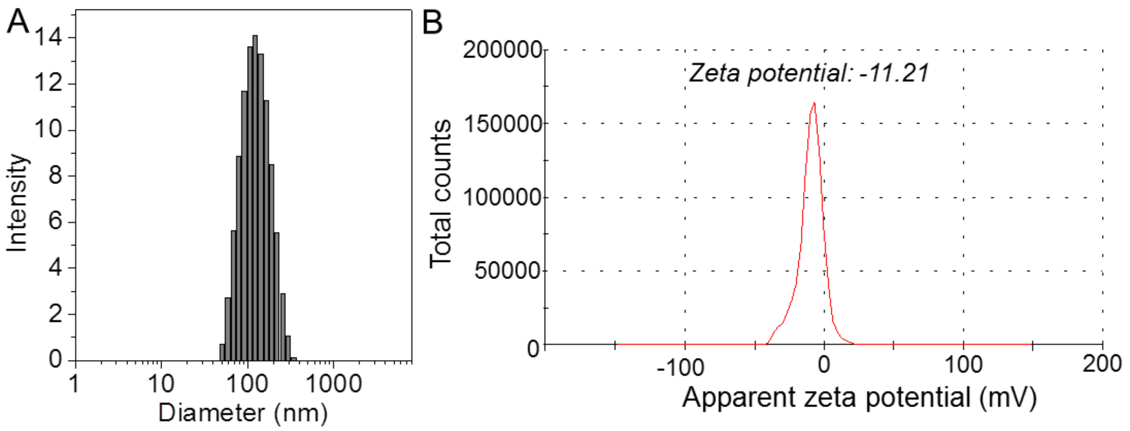

Figure S10. Size distribution (A) and zeta potential (B) of the NPs(si-1) in the aqueous solution detected by DLS.

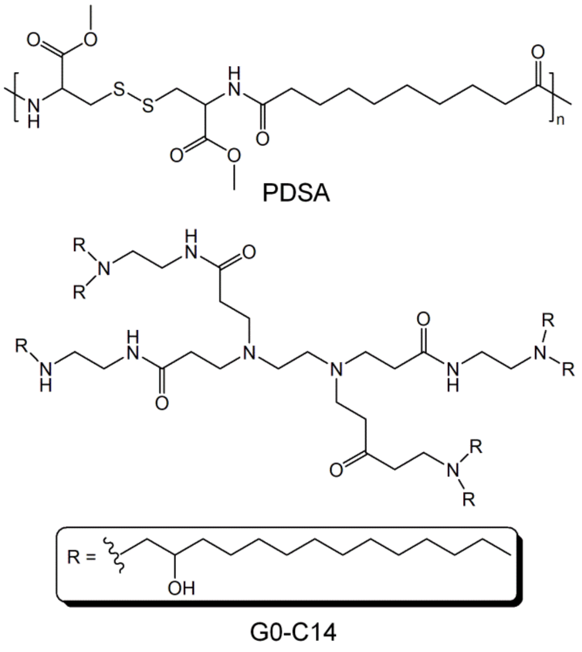

Figure S11. Chemical structures of the reduction-responsive PDSA polymer and cationic lipid G0-C14.

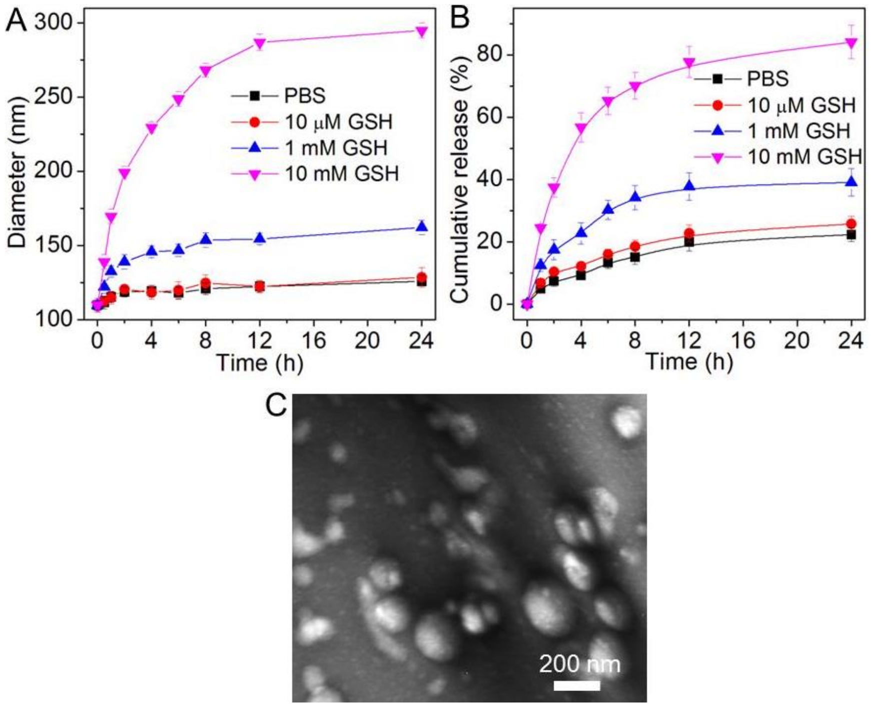

Figure S12. (A, B) Size change (A) and cumulative siRNA release (B) of the NPs(Cy5-si-1) incubated in the PBS solution with different concentration of GSH. (C) Morphology of the NPs(Ct5-si-1) incubated in the PBS solution with 10 mM GSH for 24 h.

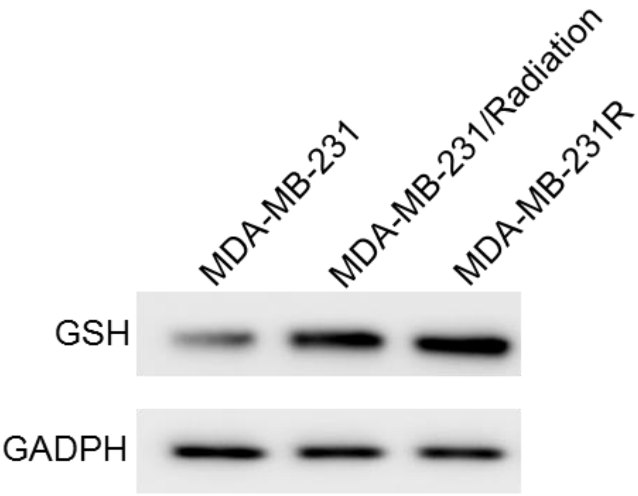

Figure S13. Western blot analysis of GSH expression in MDA-MB-231R cells and the parent MDA-MB-231 cells received 10 Gy X-ray radiation.

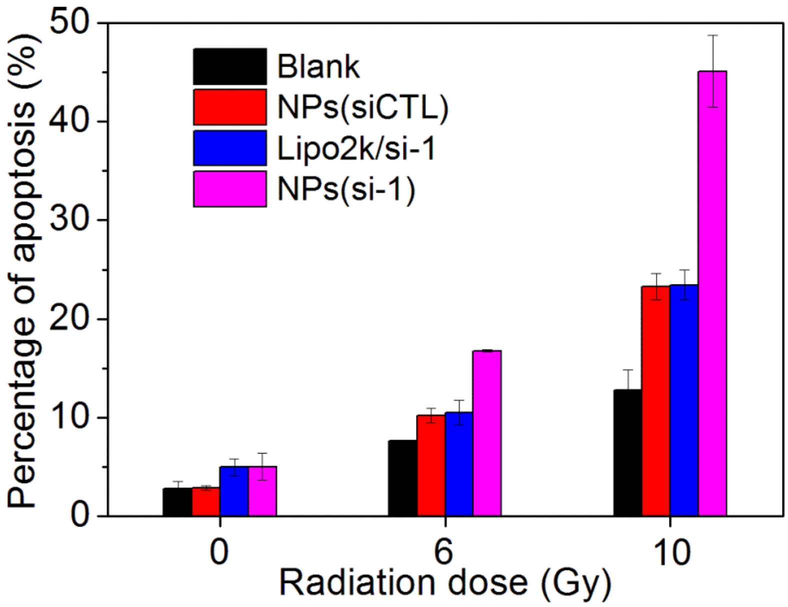

Figure S14. Percentage of apoptotic MDA-MB-231R cells treated with the Lipo2k/si-1 complexes, Lipo2k/siCTL complexes, or NPs(si-1) at a siRNA concentration of 30 nM followed by different doses of X-ray radiation.

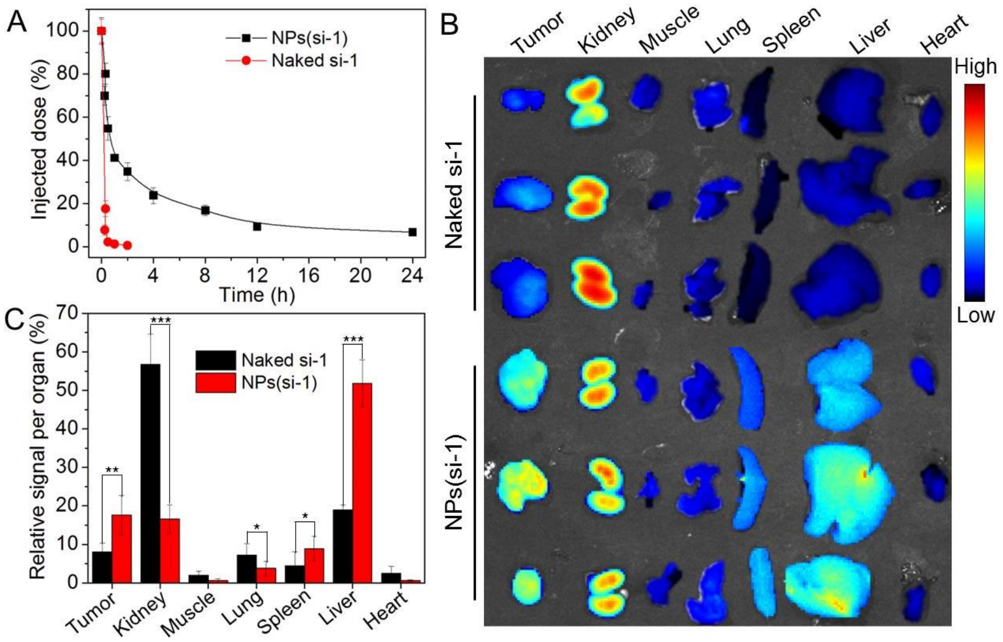

Figure S15. (A) Blood circulation profile of naked si-1 and NPs(si-1). (B) Overlaid fluorescence image of the tumors and main organs of the MDA-MB-231R xenograft tumor-bearing nude mice at 24 h post injection of naked si-1 and NPs(si-1). (C) Biodistribution of naked si-1 and NPs(si-1) quantified from (B). * $p < 0.05$, ** $p < 0.01$, *** $p < 0.001$

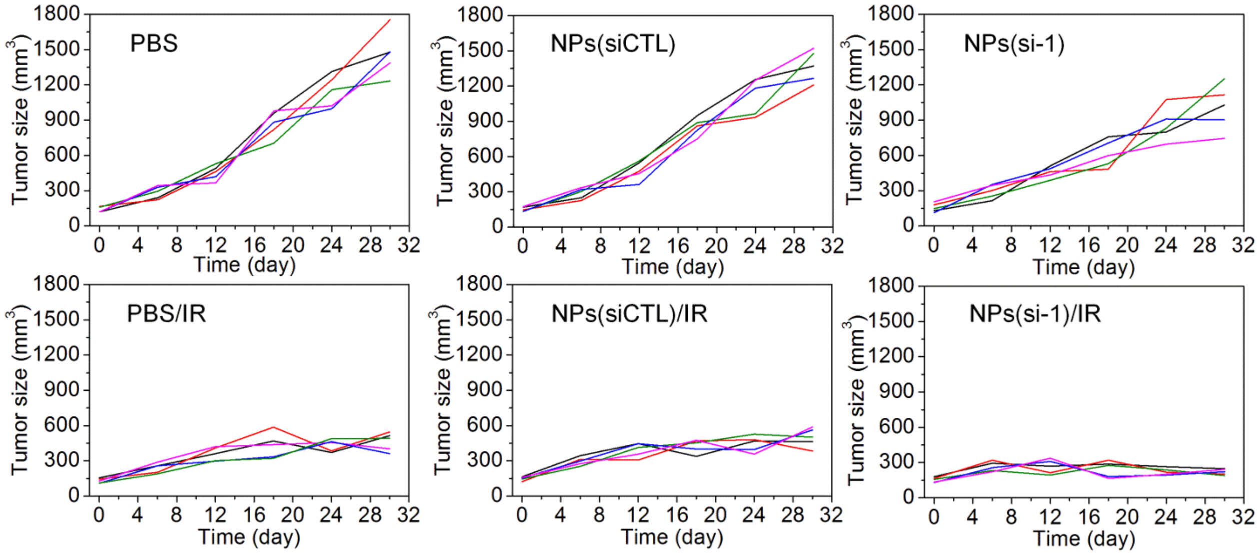

Figure S16. Tumor growth of the MDA-MB-231R tumor-bearing nude mice treated with PBS, PBS followed by ionizing radiation (PBS/IR), NPs(siCTL), NPs(siCTL) followed by ionizing radiation (NPs(siCTL)/IR), NPs(si-1), and NPs(si-1) followed by ionizing radiation (NPs(si-1)/IR) at a 1 nmol siRNA dose per mouse and 10 Gy radiation dose per mouse.

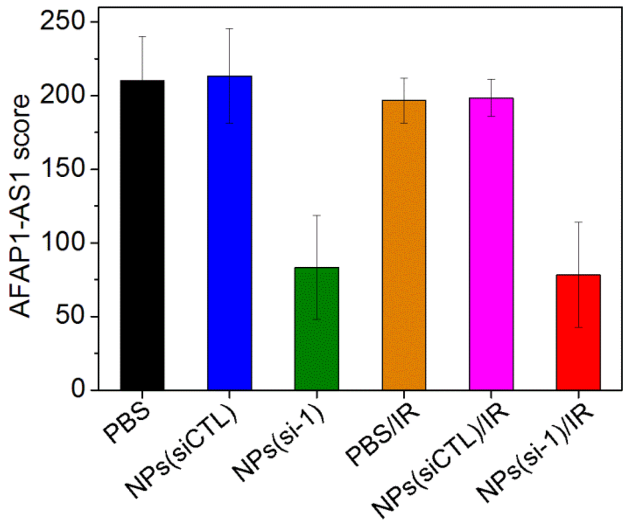

Figure S17. LncAFAP1-AS1 expression in the tumor tissues of the mice treated with PBS, PBS followed by ionizing radiation (PBS/IR), NPs(siCTL), NPs(siCTL) followed by ionizing radiation (NPs(siCTL)/IR), NPs(si-1), or NPs(si-1) followed by ionizing radiation (NPs(si-1)/IR) at a 1 nmol siRNA dose per mouse and 10 Gy radiation dose per mouse.

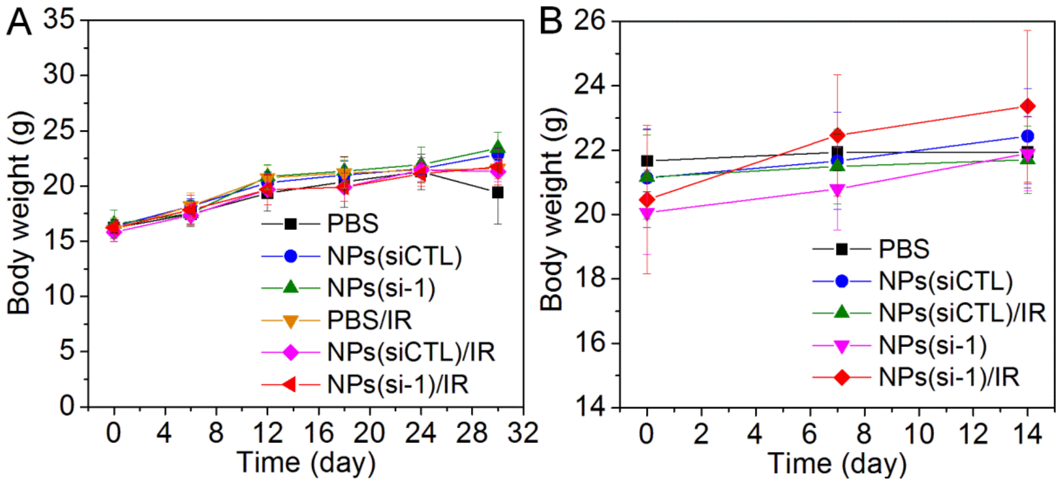

Figure S18. Body weight of the MDA-MB-231R xenograft tumor- (A) or Luc-MDA-MB-231R metastatic tumor-bearing nude mice (B) treated with PBS, PBS followed by ionizing radiation (PBS/IR), NPs(siCTL), NPs(siCTL) followed by ionizing radiation (NPs(siCTL)/IR), NPs(si-1), and NPs(si-1) followed by ionizing radiation (NPs(si-1)/IR) at a 1 nmol siRNA dose per mouse and 10 Gy radiation dose per mouse.

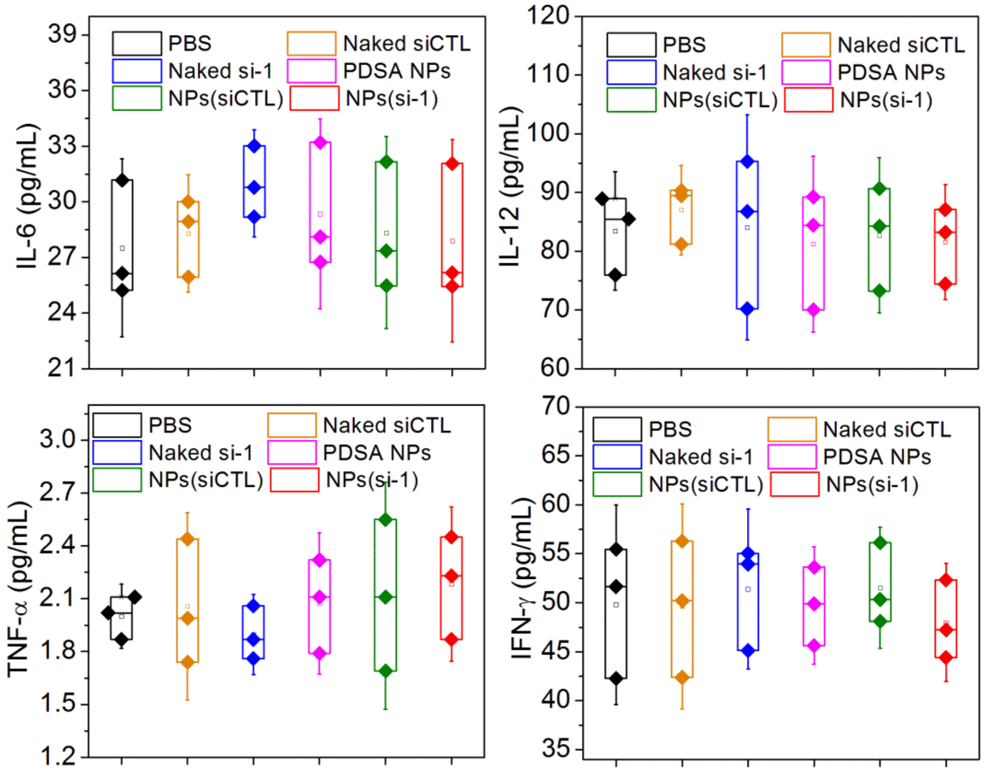

Figure S19. Serum levels of IL-6, IL-12, TNF-α, and IFN-γ at 24 h post injection of PBS, naked siCTL, naked si-1, PDSA NPs, NPs(siCTL), and NPs(si-1).

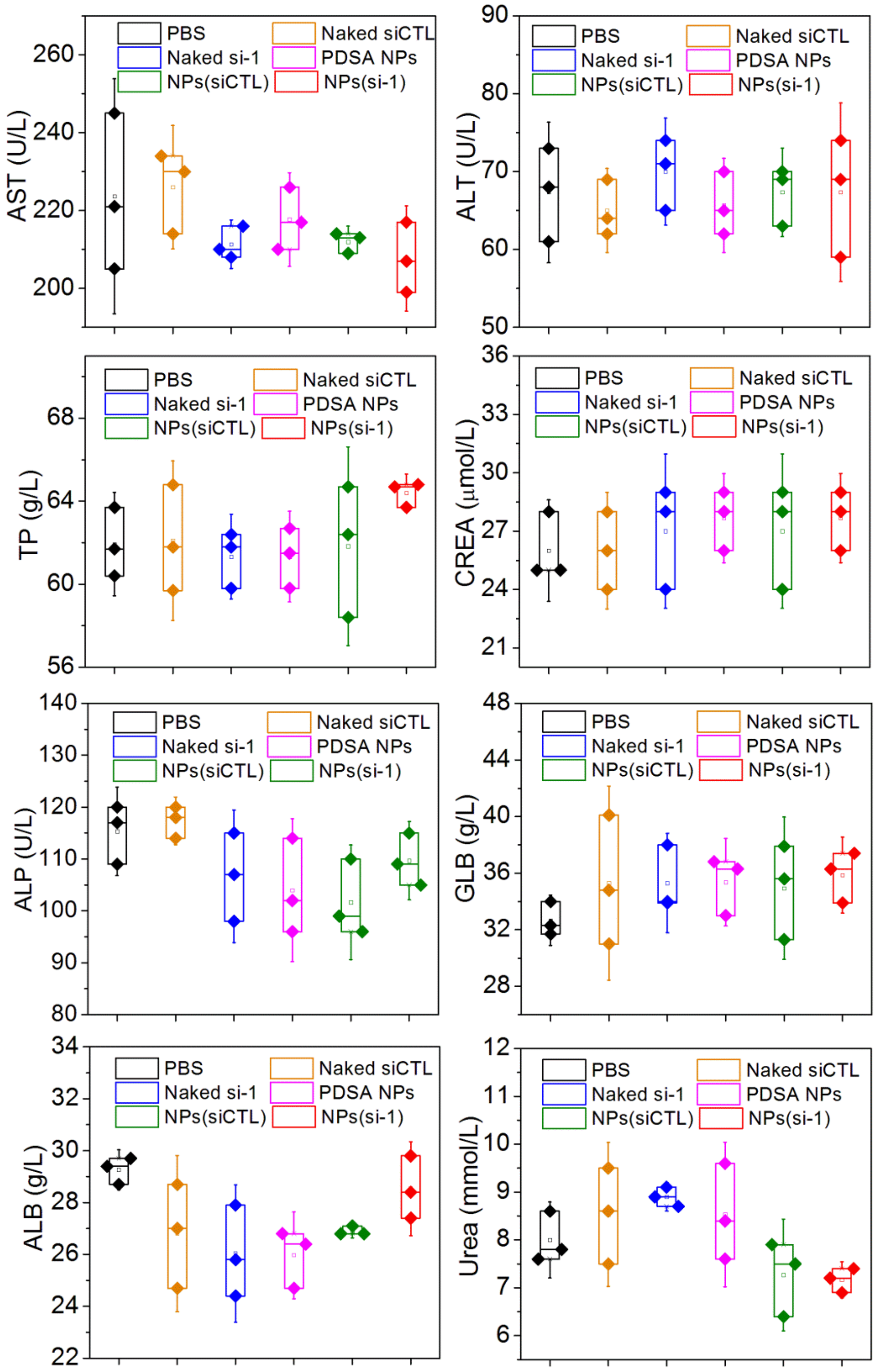

Figure S20. Serum levels of aspartate aminotransferase (AST), alanine aminotransferase (ALT), total protein (TP), creatinine (CREA), alkaline phosphatase (ALP), globulin (GLB), albumin (ALB), and urea after three consecutive injections of PBS, naked siCTL, naked si-1, PDSA NPs, NPs(siCTL), and NPs(si-1).

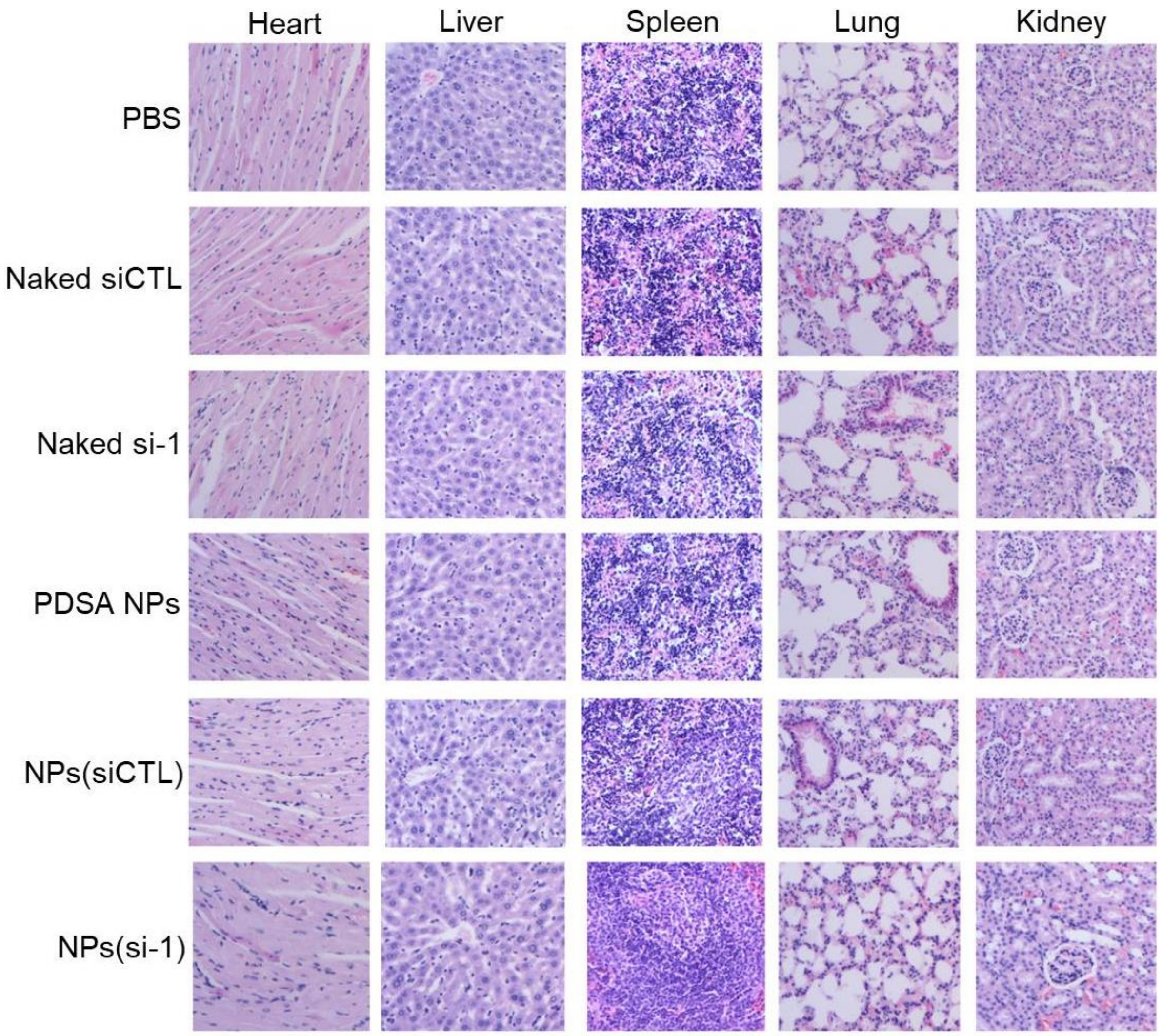

Figure S21. Histological section of the major organs after three consecutive injections of PBS, naked siCTL, naked si-1, PDSA NPs, NPs(siCTL), and NPs(si-1).
